# Dynamics of cascades on burstiness-controlled temporal networks

**DOI:** 10.1038/s41467-020-20398-4

**Published:** 2021-01-08

**Authors:** Samuel Unicomb, Gerardo Iñiguez, James P. Gleeson, Márton Karsai

**Affiliations:** 1 Université de Lyon, ENS de Lyon, INRIA, CNRS, UMR 5668, IXXI, Lyon, 69364 France; 2 Department of Network and Data Science, Central European University, Vienna, A-1100 Austria; 3 Department of Computer Science, Aalto University School of Science, Aalto, FI-00076 Finland; 4 Centro de Ciencias de la Complejidad, Universidad Nacional Autonóma de México, CDMX, 04510 Mexico; 5 MACSI and Insight Centre for Data Analytics, University of Limerick, Limerick, V94 T9PX Ireland

**Keywords:** Complex networks, Phase transitions and critical phenomena

## Abstract

Burstiness, the tendency of interaction events to be heterogeneously distributed in time, is critical to information diffusion in physical and social systems. However, an analytical framework capturing the effect of burstiness on generic dynamics is lacking. Here we develop a master equation formalism to study cascades on temporal networks with burstiness modelled by renewal processes. Supported by numerical and data-driven simulations, we describe the interplay between heterogeneous temporal interactions and models of threshold-driven and epidemic spreading. We find that increasing interevent time variance can both accelerate and decelerate spreading for threshold models, but can only decelerate epidemic spreading. When accounting for the skewness of different interevent time distributions, spreading times collapse onto a universal curve. Our framework uncovers a deep yet subtle connection between generic diffusion mechanisms and underlying temporal network structures that impacts a broad class of networked phenomena, from spin interactions to epidemic contagion and language dynamics.

## Abstract (web summary)

Temporal networks in which interaction events are distributed heterogeneously in time are complex to model. Unicomb et al. propose an analytical framework for the analysis of cascading dynamics in such networks, relevant for spin interactions, epidemic spreading, and language dynamics.

## Introduction

Temporal networks provide a representation of real-world complex systems where interactions between components vary in time^1–3^. Although initially modelled as Poisson processes, where independent events are homogeneously distributed in time, real-world networked interactions have been found to be heterogeneously distributed and to exhibit temporal correlations^4–6^. In particular, interaction events in real systems concentrate within short periods of intense activity followed by long intervals of inactivity, an effect known as burstiness. Bursty dynamics appear in diverse physical phenomena including earthquakes^7^ and solar flares^8^, biological processes like neuron firing^9^, as well as the dynamics of human social interaction^5,10^.

Burstiness in temporal interactions has profound implications for the diffusion of information over temporal networks, as demonstrated in a growing number of works^11–17^. This is true in the case of epidemic processes, often referred to as simple contagion, where the probability of infection of an uninfected node depends linearly on the number of exposures, i.e., temporal interactions with infected neighbours in the network^18^. Epidemic models successfully describe the spread of biological disease^19^, and have been shown to critically depend on burstiness and other patterns of temporal interactions^12,20–23^. Epidemic spreading over temporal networks appears to be slowed owing to burstiness in some cases^11,24–26^, whereas accelerated in others^14,27,28^. However, these conclusions are known to depend on the stage at which we observe the spreading process. It has been argued that at early stages of epidemics, short interevent times may accelerate spreading^13^, whereas at later stages, long interevent times may decelerate dynamics^16,26^. Threshold mechanisms provide another class of phenomena where bursty temporal networks have a crucial role. Threshold dynamics, also known as complex contagion, are used to model the spread of information where infection requires the reinforced influence of at least a certain fraction of neighbours in the egocentric network^29^. Threshold-driven dynamics over static networks have been extensively studied both empirically^30^ and theoretically^30–34^, but analysis of their behaviour on temporal networks is so far limited to a small number of empirical studies^35–38^. Using random reference models of temporal networks, it has been shown that when infection is driven by the fraction of infected neighbours, rather than their absolute number, bursty interactions may lead to deceleration^35,36,38^. In contrast, if the threshold measure of influence is absolute, burstiness may have an accelerative effect^35^. Acceleration has also been observed in the case of history-dependent contagion^37^.

Information diffusion in social and economic settings must overcome limits inherent to our social and cognitive capacities, namely that we have finite attention. This has motivated the concept of the attention economy^39,40^, where relative to an abundance of content and information, attention is a scarce resource. A mechanism that has emerged to deal with these limitations is ephemeral content^41,42^. Variously referred to as stories, snaps or fleets, depending on the platform, ephemeral content disappears after a specified amount of time, in principle concentrating the attention of viewers. In contrast, persistent content is not explicitly erased, but owing to cognitive limits and competing content, gradually decreases in visibility. Here, we propose an analytical framework to systematically describe the relationship between the diffusion of information, bursty temporal interactions, and inherent limits to our attention and memory, thus providing the theoretical foundation necessary to shed light on the role of burstiness in generic diffusion processes, including simple and complex contagion models of physical, biological and social phenomena.

We incorporate the most widely documented features of temporal interactions into a framework of binary-state dynamics and benchmark its behaviour with standard models of threshold driven and epidemic spreading. Although stochastic bursty interactions are likely emergent phenomena^4,43^, their dynamics are well approximated by renewal processes^44^. Temporal heterogeneity in network interactions can then be characterised by the variability in interevent times *τ*, the time between consecutive events on a given edge, parameterised by the interevent time distribution *ψ*(*τ*), whereas other features of the temporal network are considered maximally random. Renewal processes represent the simplest model of bursty, non-Markovian dynamics, and a departure from the memoryless assumption implicit in Poisson processes. Nevertheless, we are able to show that such a system can be accurately captured by a master equation formalism, which is essentially memoryless, implying the existence of a purely Markovian system with almost identical behaviour. We show both analytically and numerically that bursty temporal interactions give rise to a percolation transition in the connectivity of the temporal network, separating phases of slow and rapid dynamics for both epidemic and threshold models of information diffusion. We find that diffusion dynamics are sensitive to the choice of interevent time distribution, particularly in regard to its skewness, and we demonstrate a data collapse across distributions when controlling for this effect.

## Results

### Temporal network model

To model a temporal network, we consider an undirected, unweighted static network of *N* nodes as the underlying structure, acting as a skeleton on top of which temporal interactions take place. The degree of a node, or how many neighbours it has, takes discrete values *k* = 0, …, *N* − 1 from a degree distribution *p*(*k*). Pairwise temporal interactions, or events, occur independently at random on each static edge via a renewal process with interevent time distribution *ψ*(*τ*). Time is continuous and events are instantaneous, whereas consecutive interevent times are uncorrelated. We also assume that the renewal process is stationary (for further details, see Methods and Supplementary Note 4). By using a static underlying network, we assume that the time scales of edge formation and node addition or removal are far longer and thus negligible relative to the time scale of event dynamics over existing edges.

In its simplest form, information diffusion is a binary-state process where each node occupies one of two mutually exclusive states, which we term uninfected and infected. The probability of a node changing state is a function of the state of its neighbours, as well as the strength of their interactions. Interaction strength, also referred to as mutual influence, is a non-negative scalar that we consider to be a function of the elapsed renewal process time series. We desire that the mean of the emergent distribution of interaction strengths be stationary, and invariant to the underlying burstiness of the system. This is achieved when the contribution of a single event to interaction strength (i) goes to zero as the event ages, and (ii) is additive, meaning a spike in edge activity leads to a spike in the interaction strength between neighbours.

**Fig. 1:**
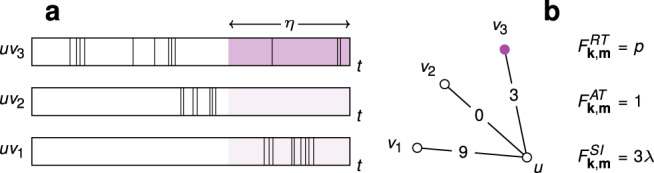
Model of information transfer via bursty temporal interactions. **a** Interaction events appear on edges according to independent renewal processes. Under a step-function memory kernel, interaction strength is determined by the number of events *j* having occurred within a time window of length *η*. Memory windows are highlighted in violet, with a darker shade indicating infection. **b** The edge-state configuration of node *u* is determined by the renewal process in **a**. Node *u* has three neighbours, with *v*_3_ being infected, so *k* = 3 and *m* = 1. The degree vector **k** of *u* contains zeros except when *j* = 0, 3 and 9, where entries equal 1. The infected degree vector **m** contains zeros, except when *j* = 3, where the entry is 1. For *v*_1_, *v*_2_ and *v*_3_, **k** contains zeros, except when *j* = 9, 0 and 3, respectively, where entries equal 1. In each case, **m** contains only zeros. The dynamical response of node *u* to its local configuration is given by the infection rate *F*_**k**,**m**_, which here assumes a relative threshold (RT case) of *ϕ* = 0.3, an absolute threshold (AT case) of *M*_*ϕ*_ = 3, and a transmission rate (SI case) of λ.

To satisfy these conditions, we are free to choose a memory kernel, or distribution of event memory times. One such coupling is a step function, i.e., the contribution of an event to the actual interaction strength is constant for a duration *η*, after which it goes to zero. This is best used to model information diffusion via ephemeral content, where the lifetime of an interaction is expressly finite. An alternative is an exponential memory kernel, where an event is forgotten at a constant rate 1/*η*, modelling persistent content with attention decaying due to other factors. For all choices of memory kernel, we define the interaction strength *w*_*j*_ of an edge at time *t* (or state *j* for short), as the number *j* of events in active memory. In the case of a step-function memory kernel, the focus of our work, it is simply the number of events in the preceding time window of width *η* (Fig. 1). Nevertheless, all studied memory kernels qualitatively lead to the same results.

It follows that the local configuration of a node is determined by the number *k*_*j*_ of its neighbours connected via edges in state *j*, with the degree *k* of the node related to its *k*_*j*_ values by *k* = ∑_*j*_*k*_*j*_ at any time *t*. We introduce *m*_*j*_ as the number of infected neighbours of a node connected via edges in state *j*. Consequently, 0 ≤ *m*_*j* _≤ *k*_*j*_ with *m* = ∑_*j*_*m*_*j*_ the total number of infected neighbours. For each node, we store *k*_*j*_ and *m*_*j*_ for all *j* in vectors **k** and **m**, providing a description of edge and node states in the local neighbourhood of a node. Nodes in class (**k**, **m**) become infected at a rate *F*_**k**,**m**_, and are statistically identical in this sense. We also store the interaction strength *w*_*j*_ = *j* in the vector **w** for all *j*. The dynamics of a given node, through *F*_**k,m**_, are thus fully determined by (**k**, **m**) and **w**.

### Information diffusion over temporal networks

To examine the effect of temporal interactions on information diffusion, we explore three widely known models of transmission. We consider both relative (RT) and absolute (AT) variants of a threshold mechanism^29,31,45^, as well as the susceptible-infected (SI) model of epidemic spreading^46^ (see Table 1 for details). All models are non-recovery, meaning the uninfected state cannot be re-entered, and we consider infection owing to external noise at a low, but non-zero rate *p*.

**Table 1:** Models of information diffusion.

RT	AT	SI
$$\left\{\begin{array}{ll}1,&{\bf{m}}\cdot {\bf{w}}\ge \phi {\bf{k}}\cdot {\bf{w}}\\ p,&{\rm{otherwise}}\hfill\end{array}\right.$$	$$\left\{\begin{array}{ll}1,&{\bf{m}}\cdot {\bf{w}}\ge {M}_{\phi }\\ p,&{\rm{otherwise}}\hfill\end{array}\right.$$	$\max (p,\,{\bf{m}}\cdot {\boldsymbol{\lambda }})$

Transmission rate *F*_**k,m**_ for nodes in configuration (**k**, **m**) with interaction strength **w** and infection rate *p* due to external noise. In complex contagion models with relative (RT) and absolute (AT) thresholds, infection is regulated by parameters *ϕ* and *M*_*ϕ*_, respectively. In the susceptible-infected (SI) model, infection is determined by the rate *λ* for a single event on an infected edge, and **λ** = λ**w** for all events on that edge. See Fig. 1 for an illustrative configuration.

The study of threshold dynamics focuses on the conditions leading to cascades, or large avalanches of infections that sweep through the network. In the simplest implementation of threshold dynamics, infection occurs when the number *m* of infected neighbours of an uninfected node exceeds a fraction *ϕ* of its degree *k*^29,31^. Generalising this rule to the case of arbitrary interaction strength^33,47^, in the RT model infection occurs when the influence of infected neighbours, **m** ⋅ **w**, exceeds a fraction *ϕ* of all potential influence, **k** ⋅ **w**. The RT model captures instances of real-world diffusion where interaction between elements affect the probability of infection only in aggregate, similar to the response of individuals to new behavioural patterns or transmission in biological neural networks^48,49^. When considering the RT model over temporal networks, the probability of infection may increase during bursts of interaction events with infected neighbours or, conversely, bursts of activity with uninfected neighbours may temporarily maintain a node in the uninfected state. This is the case in Fig. 1b, where influence from a single infected neighbour is insufficient to overcome that from uninfected neighbours. In the AT model, influence from infected neighbours is not normalised, but compared with some absolute value *M*_*ϕ*_^45^. In contrast to the RT model, infection is not hindered by interaction activity with uninfected neighbours, and bursts can only increase the probability of infection. In the SI model, finally, each interaction event with an infected neighbour triggers infection at a rate *λ*. In our framework of temporal networks, infected neighbours trigger infection via edges in state *j* at a rate *λj*. Writing ***λ*** = *λ***w**, the infection rate for a node with a neighbourhood of infected nodes described by **m** is **m** ⋅ ***λ***. Similar to the AT model, bursts can only increase the probability of infection in the SI model (Fig. 1).

### Master equation solution

We extend a master equation formalism^33,50^ to account for network temporality. We introduce the state space of all configurations (**k**, **m**) allowed by the underlying degree distribution *p*(*k*), under the condition that each edge is in one of a finite number of possible edge states (see Methods, and Supplementary Note 1 for lattice diagrams of this space). We introduce the state vector **s**(*t*) containing the probability that a randomly selected node with underlying degree *k* is uninfected and in class (**k**, **m**) at time *t*. The time evolution of **s** is governed by the matrix *W*(**s**, *t*), containing the transition rate *W*_*ij*_ from the *i*th to the *j*th configuration (**k**, **m**) at time *t*. Transitions arise from three mechanisms. First, ego transitions, contained in the matrix *W*_ego_, describe the loss to configuration (**k**, **m**) owing to its nodes becoming infected. This occurs at a rate *F*_**k**,**m**_, as per Table 1, so the diagonal terms of *W*_ego_ are given by  −*F*_**k**,**m**_ and off-diagonals are zero. Second, neighbour transitions, contained in matrix *W*_neigh_, describe the gain or loss to configuration (**k**, **m**) owing to the infection of neighbours of nodes related to this class. This transition is determined by *β*_*j*_*dt*, the probability of an uninfected neighbour in configuration *j* becoming infected over an interval *dt* (see Methods for an explicit calculation). Taken together, *W*_ego_ and *W*_neigh_ accurately describe diffusion dynamics over static networks with heterogeneous edge types, such as weighted and multiplex networks^33,34^.

Temporal networks require a third component, edge transitions, contained in the matrix *W*_edge_, describing the gain or loss to configuration (**k**, **m**) due to changes in an edge’s state *j*. This applies to any temporal network model that can be formulated in terms of discrete, dynamic edge states. We denote by *μ*_*j*_*dt* and *ν*_*j*_*dt* the probabilities that a randomly selected edge in state *j* undergoes a positive or negative transition and enters state *j* + 1 or *j* − 1, respectively, over an interval *dt*. Combining these terms gives the master equation

1 $$\frac{d}{dt}{\bf{s}}=({W}_{{\rm{ego}}}+{W}_{{\rm{neigh}}}+{W}_{{\rm{edge}}}){\bf{s}}=W({\bf{s}},t){\bf{s}}.$$

Modelling temporal network dynamics amounts to solving Eq. (1), which along with the initial condition **s**(0), determines the evolution of the system.

To apply this formalism we derive the edge transition rates *μ*_*j*_ and *ν*_*j*_ in the case of renewal processes. We first note that microscopically, on the scale of a single edge, transitions from state *j* to *j* ± 1 cannot be described by a constant rate. In a renewal process, the probability of an event occurring is conditional on the time elapsed since the previous event. Therefore, this probability is history dependent, meaning edges have an effective memory and are non-Markovian by definition. Further, since it is only the previous event that is determinant, there is clearly no *j* dependence at this scale. A renewal process may then seem at odds with a Markovian master equation [where **s**(*t* + *dt*) depends only on **s**(*t*), as per Eq. (1)]. Macroscopically, however, on the scale of large ensembles of edges, the renewal process exhibits effective *j*-dependent rates that are constant in time. We can calculate the probability *E*_*j*_ that a randomly selected edge is in state *j*, and the probability that it transitions to state *j* ± 1 over an interval *dt*, giving *μ*_*j*_ and *ν*_*j*_ [see Methods for explicit expressions for *j* > 0, with the *j* = 0 case of *E*_*j*_ and *μ*_*j*_ comprising a special case that we define in Eqs. (2) and (3) below].

As the rates *μ*_*j*_ and *ν*_*j*_ are heterogeneous in terms of *j*, they can be viewed as a signature of the model parameters *ψ*(*τ*) and *η*, and of the non-Markovianity inherent at the scale of a single edge. On a macroscopic scale, *μ*_*j*_, *ν*_*j*_, and *E*_*j*_ are constant in time, meaning our system is indistinguishable from a continuous-time Markov chain model of edge state. That is, a random walk on the non-negative integers, with transition rates given by *μ*_*j*_ and *ν*_*j*_, and a stationary distribution of walkers given by *E*_*j*_ (see Supplementary Note 2 for an illustration of *μ*_*j*_ and *ν*_*j*_ in the case of gamma-distributed interevent times). Applying the system-wide rates *μ*_*j*_ and *ν*_*j*_ at the finer-grained level of configurations (**k**, **m**) amounts to a mean field approximation. Monte Carlo simulations (see Supplementary Fig. 10) demonstrate that the actual edge transition rates deviate slightly from *μ*_*j*_ and *ν*_*j*_ for each  class (**k**, **m**), even if they are exact for the network as a whole, in the limit of large *N*. The accuracy of the master equation solution provides a measure of the remarkable similarity between a renewal process, where Eq. (1) is an approximation, and the biased random walk interpretation of edge state, where Eq. (1) is exact.

### Information diffusion and burstiness

**Fig. 2:**
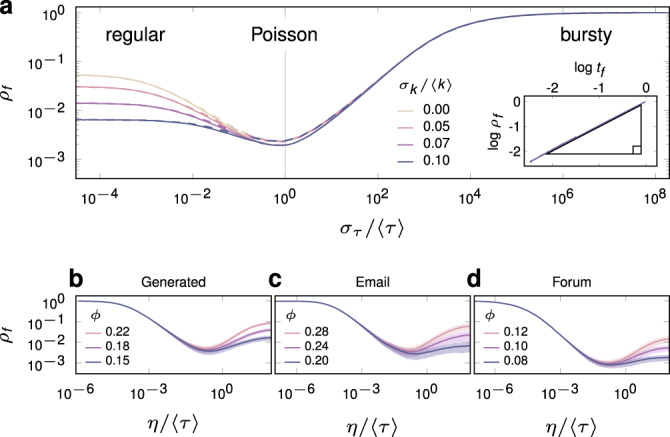
Fraction of infections due to noise, *ρ*_*f*_, as a function of interevent time distribution. Normalised diffusion time *t*_*f*_ produces an almost identical effect (see **a**, inset). The small *σ*_*τ*_ limit, leading to regular patterns in *τ*, comprises the quenched limit in **a** where *η* = 1 and the network is effectively static. The large *σ*_*τ*_ limit produces large bursts in activity, comprising the annealed regime where the network is effectively sparsified and plays no role in information diffusion (*ρ*_*f*_ = 1). Mirroring results are achieved by varying memory *η* for fixed *σ*_*τ*_ in generated, **b**, and empirical, **c**–**d**, temporal networks. Analytic solution is denoted by dashed lines, and Monte Carlo results by solid lines. Generated networks have lognormal degree distribution with mean 〈*k*〉 = 7 and standard deviation *σ*_*k*_ = 2. We use Weibull-distributed interevent times with mean 〈*τ*〉 = 1. Plot **b** uses *σ*_*τ*_ = 1. For empirical data description see Methods. Node dynamics correspond to the RT model with threshold *ϕ* = 0.15 and external noise *p* = 2 × 10^−4^. Cutoff density is *ρ*_*c*_ = 0.4. Monte Carlo simulations are averaged over 10^4^ realisations. Network size is 10^6^ in **a**, and 5 × 10^3^ in **b**.

We validate our analytical framework with Monte Carlo simulations of diffusion dynamics over temporal networks. Simulations use an underlying static, configuration-model network with lognormal degree distribution of mean 〈*k*〉 and standard deviation *σ*_*k*_. We measure the time *t*_*c*_ required to reach an arbitrary density *ρ*_*c*_ of infected nodes, in the presence of background noise at rate *p*. We also measure *ρ*_*f*_, the relative frequency of infections due to external noise, such that 0 < *ρ*_*f*_ ≤ 1, with 1/*ρ*_*f*_ the ratio of total to noise-induced infections, measuring the catalytic effect of external noise (for a detailed description of *ρ*_*f*_ see Methods). We normalise *t*_*c*_ by the time taken to reach the desired density by noise alone, providing *t*_*f*_, such that 0 < *t*_*f*_  ≤ 1 . Remarkably, *ρ*_*f*_ and *t*_*f*_ are close to linearly proportional (Fig. 2a, inset). A value of *ρ*_*f*_ = *t*_*f*_ = 1 indicates slow diffusion with complete reliance on external noise, whereas small *ρ*_*f*_ and *t*_*f*_ represent rapid diffusion with external noise producing a substantial catalytic effect. Together, they measure the extent to which the temporal network, rather than external noise, drives the diffusion of information.

We first examine the effect of varying interevent time standard deviation *σ*_*τ*_ for fixed memory *η* = 〈*τ*〉 = 1 (Fig. 2a). We choose a Weibull interevent time distribution *ψ*(*τ*), used widely to model behavioural bursts in both human^51^ and animal^52^ dynamics. A Weibull distribution reduces to the exponential distribution when  *σ*_*τ*_ = 〈*τ*〉 = 1. Node dynamics follow the RT model with threshold *ϕ* = 0.15 and background noise *p* = 2 × 10^−4^. Approaching the small *σ*_*τ*_ limit from above, events arrive in an increasingly regular pattern, and an increasing fraction of edges are frozen in the mean state *η*/〈*τ*〉 = 1. We refer to this as the quenched regime, whereby edges converge to a single state and the network is effectively static. In the opposing limit of large *σ*_*τ*_, burstiness means that at any given time, edge activity is concentrated among an arbitrarily small fraction of edges that undergo large spikes in activity, with the remainder in state *j* = 0. We refer to this as the annealed regime, where the network is maximally sparse and has a vanishingly small role in information diffusion (*ρ*_*f*_ and *t*_*f*_ approach one).

Both quenched and annealed regimes lead to slow, noise-reliant diffusion, with the expected edge state *η*/〈*τ*〉 preserved in each case (Fig. 2a). For intermediate values of *σ*_*τ*_, there is a well-mixed regime where relatively rapid diffusion occurs, owing to edge-state fluctuations that are ultimately favourable to transmission. In the RT model this implies a spike of activity on an infected neighbour overcoming a node’s threshold, or decreased activity on uninfected edges lowering the relative influence to be overcome. The decelerative effect of quenching is increased for narrower underlying degree distributions, as an increasing fraction of nodes are frozen in a state unfavourable to transmission, a static network effect already reported in ref. ^31^.

As seen in Fig. 2a, if the system is more bursty than a Poisson process, i.e., if *σ*_*τ*_ ≥ 1, values of *ρ*_*f*_ coincide regardless of the underlying degree distribution. The degree distribution is degenerate in this sense, as temporal fluctuations, rather than the underlying degree, determine local connectivity over short time scales. In Supplementary Note 6, we show that clustering is another such property, where even densely clustered configuration-model networks^53^ are functionally treelike in the annealed phase, with long interevent times *τ* inducing edges to the non-interacting state, thereby deconstructing cycles. We conjecture that local properties, like node degree and clustering, are degenerate in the presence of uncorrelated temporal fluctuations, whereas collective properties like modularity and eigenvector centrality are not. In Supplementary Fig. 9 we demonstrate using the Watts-Strogatz model^54^ that average path length, a manifestly collective property, is clearly non-degenerate. Interestingly, these results suggest that temporal correlations between edges on a local scale may break the degeneracy observed in Fig. 2a. The study of such correlations is an active line of research^55^.

A mirroring effect can be obtained by varying memory *η* for constant *σ*_*τ*_ = 〈*τ*〉 = 1 (Fig. 2b). The quenched limit is recovered for large *η*, as large samples of events on each edge result in edges converging to a mean state, *η*/〈*τ*〉, with an increasingly narrow distribution, owing to the central limit theorem. As for the case of fixed *η*, quenching may be decelerative if cascades on the corresponding static network are noise dependent. For example, increasing *ϕ* can cause slower diffusion in the quenched limit (Fig. 2b). The annealed, or noise-reliant regime is effectively recovered when *η* is vanishingly small, meaning almost all edges are in state *j* = 0 and the role of the network in information diffusion vanishes (*ρ*_*f*_ = *t*_*f*_  = 1). The correspondence between *σ*_*τ*_ and *η* suggests data-driven experiments that allow an indirect inference of the effects of varying *σ*_*τ*_ in real systems, an open problem in the study of information diffusion. We simulate the RT model on two empirical temporal networks and vary only the memory *η*, recovering qualitatively the effects observed on synthetic networks (Fig. 2c, d, see Methods for data description). This suggests the accelerative and decelerative effects of burstiness may well be a feature of real-world information diffusion.

### Comparing interevent time distributions

**Fig. 3:**
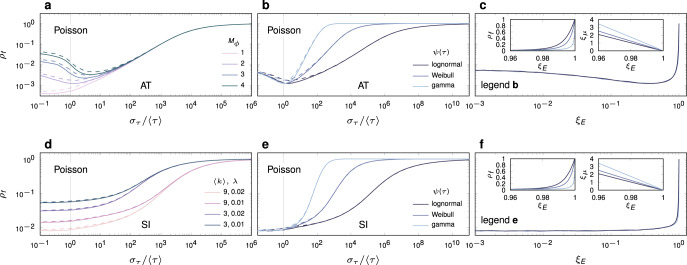
Fraction of infections due to noise, *ρ*_*f*_, for various choices of interevent time distribution *ψ*(*τ*). Information diffuses following the AT, or absolute threshold model, **a**–**c**, and the SI, or susceptible-infected model, **d**–**f**. Analytic solution is denoted by dashed lines, and Monte Carlo results by solid lines. **a** Effect of absolute threshold *M*_*ϕ*_. **b** Dependence of *ρ*_*f*_ on choice of *ψ*(*τ*). **c** Data collapse of **b** after controlling for effective sparsity *ξ*_*E*_. Left inset is a closeup of the main plot in linear scale, revealing that differences in *ρ*_*f*_ remain after controlling for *ξ*_*E*_. This is explained by the differing mixing rates *ξ*_*μ*_ (inset right). **e**, **f** Corresponding results for the SI model. Cutoff node density is *ρ*_*c*_ = 0.4, with *η* = 〈*τ*〉 = 1. Analytic solution is shown by dashed lines, Monte Carlo simulations by solid lines. Degree distribution is lognormal with 〈*k*〉 = 7 and *σ*_*k*_ = 1. We use a threshold *M*_*ϕ*_ = 2 for the AT model, $\lambda = 0.02$ in the SI model, and external noise *p* = 2 × 10^−4^ in each. Monte Carlo simulations involve 10^4^ realisations on networks of size *N* = 10^6^.

Next, we compare the dynamical outcomes of diffusion under lognormal, Weibull and gamma interevent time distributions. Each of these are two-parameter distributions, whose values we determine by specifying their mean 〈*τ*〉 and standard deviation *σ*_*τ*_. Consider first the AT model with *M*_*ϕ*_ = 2 and *η* = 〈*τ*〉 = 1 (Fig. 3b). Here, we observe a striking dependence of diffusion dynamics on *ψ*(*τ*), with the lognormal distribution leading to the most rapid diffusion, outpacing the gamma distribution in diffusion speed and relative noise dependence by up to a factor of 83, and the Weibull distribution by up to a factor of 14. Consider too that in order to achieve an identical dynamical outcome as the gamma distribution, say *ρ*_*f*_ = 0.1, the standard deviation of the lognormal distribution must be increased by over three orders of magnitude. These dramatic differences can be accounted for by comparing the rate of onset of annealing in terms of the fraction of edges in state *j* = 0, as we increase *σ*_*τ*_. To quantify this effect, we introduce

2 $${\xi }_{E}=\int_{\eta }^{\infty }\Psi (\tau )d\tau$$

and

3 $${\xi }_{\mu }=\frac{\mathop{\int}\nolimits_{\eta }^{\infty }\psi (\tau )d\tau }{\mathop{\int}\nolimits_{\eta }^{\infty }\Psi (\tau )d\tau },$$

where *ξ*_*E*_ equals *E*_0_, the fraction of edges in state zero, and *ξ*_*μ*_ equals *μ*_0_, the probability that a randomly selected edge in state zero enters state one over an interval *dt*. We may refer to *ξ*_*E*_ as the effective sparsification, or alternatively, the effective annealing. Here Ψ(*τ*) is the complementary cumulative distribution relating to *ψ*(*τ*). The gamma distribution rapidly anneals the network, yielding the largest *ξ*_*E*_ values of all choices of distribution, meaning the most edges in state *j* = 0. As a result, it exhibits the slowest, most noise-reliant diffusion. In terms of the value of *ξ*_*E*_ induced, the gamma is followed by the Weibull distribution, then the lognormal distribution. In fact, the lognormal requires orders-of-magnitude larger *σ*_*τ*_ to produce equal values of *ξ*_*E*_ as the Weibull and gamma distributions. By plotting *ρ*_*f*_ against *ξ*_*E*_ we observe the data to collapse approximately onto a single curve, revealing *ξ*_*E*_ to be a far better predictor of dynamics than *σ*_*τ*_ (see Fig. 3c in contrast to Fig. 3b). Some disagreement persists, however (Fig. 3c, left inset), which can be explained by noting that increased rates of mixing *ξ*_*μ*_ (Fig. 3c, right inset) ensure that the small number of active edges redistribute about the network at a greater rate, thus mediating cascades more effectively. An identical effect is observed for the SI model (Fig. 3e, f).

The data collapse in Fig. 3c, f confirm that above all it is *ξ*_*E*_, the density of edges in state *j* = 0, that ultimately determines the diffusion dynamics in our framework. The sensitivity of *ξ*_*E*_ to the choice of interevent time distribution *ψ* can be seen in Eq. (2), where the dependence is functional. We wish to go further, however, and identify the properties of a given distribution *ψ* that contribute to the value of *ξ*_*E*_, beyond its mean and standard deviation. We explore these properties using the generalised gamma distribution in the following section.

### Temporal network phase transition

**Fig. 4:**
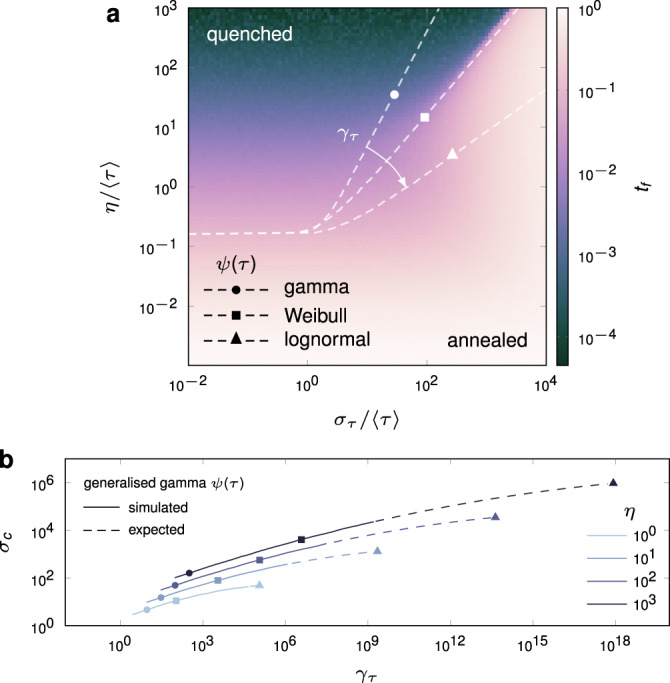
Collapse of the giant temporal component due to increasing burstiness. **a** Heat map is diffusion time *t*_*f*_ for an SI process with *λ* = 0.02 under a Weibull interevent time distribution, and a step-function memory kernel. The underlying degree distribution is lognormal with 〈*k*〉 = 7 and *σ*_*k*_ = 0.5, and network size *N* = 10^5^. The slope of the temporal network phase transition, dashed lines, depends on the choice of the interevent time distribution, and can be parameterised by its skewness, *γ*_*τ*_. **b** For a given *η*, the critical *σ*_*c*_ varies for the gamma, Weibull and lognormal distributions, which are special and limiting cases of the generalised gamma distribution, parameterised here by *γ*_*τ*_. Solid lines in **b** result from simulating the temporal network with smoothly varying *γ*_*τ*_ under the generalised gamma distribution. Symbols are data taken from **a**, the results of direct simulation of the gamma, Weibull and lognormal distributions. Dashed lines are extrapolation from solid lines to lognormal data.

We systematically explore (*σ*_*τ*_, *η*) space using Monte Carlo simulations of the SI model (Fig. 4). We aim first to understand how the temporal connectivity evolves as a function of *σ*_*τ*_ and *η*. As previously observed, the quenched regime appears either in the small *σ*_*τ*_ limit for constant (but sufficiently large) *η*, or in the large *η* limit for constant *σ*_*τ*_. The temporal network enters the annealed regime in two ways, either by taking the small *η* limit for constant *σ*_*τ*_, or the large *σ*_*τ*_ limit for constant *η*. The two regimes are separated by an edge percolation transition, i.e., the emergence of a giant connected component in the sub-graph formed by edges in state *j* > 0, that can be expected with high probability at any given time (see regimes and boundary in Fig. 4a). The giant component is dynamic, meaning its composition in terms of both nodes and edges is constantly evolving, but can always be expected to percolate through the network. Above percolation, a giant components facilitates the diffusion of information, that would otherwise rely on small, temporally disconnected components in order to propagate. We denote by *q*(*k*) the degree distribution obtained by randomly removing a fraction *ξ*_*E*_ of edges in a static configuration-model network with degree distribution *p*(*k*), which is identical to that of the expected sub-graph formed by removing state zero edges in the stochastic temporal network. The percolation transition for *q*(*k*) can be computed analytically (see Supplementary Note 3), and despite the static assumption, provides an excellent estimate of the boundary between quenched and annealed regimes of the temporal network (Fig. 4a), indicating the onset of slow, noise-dependent diffusion for all diffusion dynamics considered.

The percolation transition in Fig. 4a is heavily dependent on *ψ*, as seen in the angle formed in the (*σ*_*τ*_, *η*) plane by the gamma, Weibull and lognormal interevent time distributions (left to right). For a given value of memory *η*, we denote by *σ*_*c*_ the critical value of interevent time standard deviation, indicating the collapse of the giant temporally connected component. To appreciate the importance of the choice of *ψ*, consider that when memory is set to *η* = 10, the collapse of the giant component occurs at *σ*_*c*_ = 15 under the gamma distribution, then orders-of-magnitude later at 80 and 1300 under the Weibull and lognormal distributions, respectively. Above all, these results emphasise that for an arbitrary interevent time distribution, mean and standard deviation alone are insufficient to estimate dynamical quantities, such as temporal connectivity.

To better understand the behaviour of the temporal network phase transition, critical values of interevent time standard deviation, *σ*_*c*_, are drawn from Fig. 4a and plot as symbols in Fig. 4b. We do this for select values of memory, *η*. Then, we exploit the fact that the distributions in Fig. 4a are either special (gamma and Weibull) or limiting (lognormal) cases of the generalised gamma distribution, a three-parameter distribution that may be used to model interevent times directly, or be used to discriminate between two-parameter models in empirical settings^56^. We specify two of the three parameters in the generalised gamma distribution using 〈*τ*〉 and *σ*_*τ*_, leaving a third parameter that may be used to control a number of higher order properties, such as the interevent time skewness, *γ*_*τ*_. Other parameterisations are possible, such as differential entropy, defined in Supplementary Note 8. The solid curves in Fig. 4b give the critical point *σ*_*c*_ observed when smoothly varying *γ*_*τ*_ in the generalised gamma distribution. As expected, *σ*_*c*_ in the simulated network interpolates between that of the gamma and Weibull distributions. The lognormal distribution is numerically challenging to recover in this manner, being a limiting case, but in principle belongs to the same family. The dashed line is the extrapolation to this case.

The variation between phase transitions in Fig. 4a appears to be characterised by a single parameter, namely the angle formed in the (*σ*_*τ*_, *η*) plane. This intuition is validated by the generalised gamma distribution, whose third parameter, specified here by interevent time skewness *γ*_*τ*_, accounts for this effect. Skewness should be viewed here as a proxy of higher order structure, as a number of other quantities, such as differential entropy, vary alongside *γ*_*τ*_. A consequence of this may be the following. Given a set of empirical interevent time data, measurements of its mean and standard deviation alone are insufficient to infer its dynamics, such as spreading time (as in Fig. 3b, d), or whether the temporal network is above percolation, below percolation, or at criticality (as in Fig. 4a). Figure 4 demonstrates that performing just one additional measurement, such as the skewness of the *τ* data, is sufficient to characterise the temporal connectivity, and consequently, the diffusion dynamics.

These results are unchanged qualitatively when the step-function memory kernel is replaced with an exponential kernel with mean *η*. The difference lies in the slope of the transition in the (*σ*_*τ*_, *η*) plane, with the giant component proving more robust everywhere in the exponential case (see Supplementary Fig. 12). For instance, when *η* = 10, the giant component collapses at *σ*_*τ*_ = 80 under a step-function memory kernel, but not until 300 under an exponential kernel. Although a substantial effect, it is small relative to that of varying interevent time skewness. Further, since the basic connectivity of the temporal network is determined by *ψ* and *η*, the outcome of information diffusion (as measured by *t*_*f*_ in Supplementary Fig. 7) is qualitatively similar across diffusion models. In particular, below percolation, all transmission events take place in small temporally disconnected components, that appear and disappear dynamically. Above percolation, the diffusion process is accelerated thanks to the giant connected component. The percolation transition is clear regardless of one’s choice of diffusion model.

## Discussion

Our study shows that generic dynamics of information diffusion are closely tied to the level of burstiness in the underlying temporal network. By considering three binary-state models of transmission, we have demonstrated that they differ in their response to burstiness only in their details. For instance, while having a purely decelerative effect on SI models, increasing burstiness at intermediate values can be accelerative for threshold models. Nevertheless, the prevailing trend suggests that increasing burstiness is strongly decelerative overall, with the onset of the decelerative phase heavily dependent on the choice of the interevent time distribution. The key assumptions here are that the underlying network is fixed, and that owing to a memory mechanism, a fraction of edges enter a non-interacting state owing to long waiting times. These assumptions result in a temporal network topology that has profound implications for many dynamical processes. It is likely that structural features of the temporal network, such as the percolation transition separating slow and fast diffusion, will also be critical for the more general class of binary-state dynamics.

Indeed, one of the strengths of our master equation formalism is that it is straightforward to extend to general binary-state models of node dynamics. This includes not only threshold and epidemic models, but language, voter, and Ising models, among others (see Supplementary Note 1). Our framework can also accommodate a broad class of temporal network models. In particular, any model that can be formulated in terms of discrete, dynamic edge states is a candidate for our approach. This includes growing, decaying and adaptive networks, as well as models of rewiring. In line with our use of renewal processes, a large family of point processes have natural descriptions in terms of discrete edge state, such as cascading Poisson, Cox and Hawkes processes. Extensions to the Poisson process in general represent promising applications of our methodology. The main limitation of our approach, however, is the sheer size of the master equation solution, in that it can be just as fast to simulate the dynamics as to solve its equations numerically. In particular, note that we did not solve the system at large values of memory *η*, as this entails a large average edge state, and a prohibitively large configuration space.

Although a master equation is unlikely to be one’s first choice of tool when analysing empirical temporal network data, the by-products of a master equation study are immediately applicable. In particular, our study highlights the importance of moments beyond mean and standard deviation when fitting interevent time distributions. We have used the generalised gamma distribution to interpolate between the lognormal, Weibull and gamma distributions, and in so doing, demonstrated a remarkable sensitivity of dynamic quantities such as the temporal network percolation transmission. Estimating the connectivity of empirical systems, for example, by accounting for interevent time skewness, is one intriguing application of this technique. Further, our treatment of non-Markovianity may be applied to other systems. That is, while a single component in a large system may be strongly non-Markovian, as was the case in our renewal process, stationary statistics may emerge at an ensemble level that act as a signature of the non-Markovianity in play microscopically. Our biased random walk interpretation of the renewal process model shows that strikingly similar Markovian counterparts may be available for analysis. Incidentally, biased random walk models of edge state suggest a broad class of Markovian models to which our master equation applies exactly. Markovian systems may then be used in this way as a probe of various complex systems where memory is critical.

## Methods

### Master equation configuration space

We provide here an outline of the master equation formalism. In this work, our approach has been to assign one of a finite number of types to each edge, and to allow this quantity to evolve over time. To formulate a master equation solution, one defines a state space of allowed node configurations, which we term configuration space. The second step is to define the allowed transitions between node configurations. The time evolution of a probability density over this state space amounts to a set of first-order differential equations, or rate equations, that among other things, provides the total density of infected nodes at a given time.

First, we define *C*_**k**,**m**_, the set of all nodes in the network with local configuration (**k**, **m**), such that 0 ≤ *m*_*j*_ ≤ *k*_*j*_, for all *j*. Whereas *C*_**k**,**m**_ is a set of nodes, we define *C*_*k*_ as the set of all sets *C*_**k**,**m**_ with total degree *k*. In a similar way we define the configuration space *C* as the set of all possible sets *C*_**k**,**m**_, given a degree distribution *p*(*k*). These sets can be written

4 $${C}_{k}=\left\{{C}_{{\bf{k}},{\bf{m}}} \mid \sum_{j}{k}_{j}=k\;{\rm{and}}\;0\le {m}_{j}\le {k}_{j}\right\}$$

and

5 $$C=\bigcup _{k}{C}_{k},$$

where the union is over all *k* in the support of *p*(*k*). Importantly, *C* leads to a partition of the network at any given time. Written this way, *C* is potentially infinite. Imposing the constraint that it be finite, we assume an upper cutoff in the degree distribution *p*(*k*), and the set of edge states to be of a finite size *n*. Note that *C* includes any set for which *C*_**k**,**m**_ is empty at a given time. The cardinality ∣*C*∣ of configuration space is thus determined entirely by the support of *p*(*k*), along with *n*. Since (**k**, **m**) does not convey ego state, just edge and neighbour configuration, we partition *C*_**k**,**m**_ into sets of uninfected and infected nodes, such that *C*_**k**,**m**_ = *S*_**k**,**m**_ ∪ *I*_**k**,**m**_. Similar definitions allow us to introduce *S*_**k**_ and *I*_**k**_, *S*_*k*_ and *I*_*k*_, as well as *S* and *I*. Although in general ∣*S*_**k**,**m**_∣ ≠ ∣*I*_**k**,**m**_∣, the structure of the uninfected and infected configuration spaces is identical, such that ∣*C*∣ = ∣*S*∣ = ∣*I*∣, ∣*C*_*k*_∣ = ∣*S*_*k*_∣ = ∣*I*_*k*_∣ and ∣*C*_**k**_∣ = ∣*S*_**k**_∣ = ∣*I*_**k**_∣.

### Density of states

The evolution of a dynamical process over a network amounts to a flow of nodes through the sets *S*_**k**,**m**_ and *I*_**k**,**m**_ over time. As the number of nodes *N* in the network is conserved, it is their distribution over the sets *S*_**k**,**m**_ and *I*_**k**,**m**_ that evolves in time. These distributions provide the state of the process at time *t*. As our formalism is independent of network size, we deal with the densities of nodes rather than the absolute sizes of these sets. To this end, we introduce

6 $$\parallel {C}_{k}\parallel \ \equiv \sum _{{C}_{{\bf{k}},{\bf{m}}}\in {C}_{k}}| {C}_{{\bf{k}},{\bf{m}}}|$$

as shorthand for the number of nodes with underlying degree *k*. This is in contrast to ∣*C*_**k**_∣ and ∣*C*_*k*_∣, which give the number of configurations with degrees **k** and *k*, respectively. To convert from absolute node count to  node density, we need to normalise *S*_**k**,**m**_ and *I*_**k**,**m**_ by some non-zero quantity that is conserved over the course of a dynamical process. As our temporal network models assume a static underlying network, a node’s underlying degree *k* is preserved, and as a result, so is ∥*C*_*k*_∥, defined in Eq. (6). The density of uninfected nodes in class (**k**, **m**) in this case is given by

7 $${s}_{{\bf{k}},{\bf{m}}}=\frac{| {S}_{{\bf{k}},{\bf{m}}}| }{\parallel {C}_{k}\parallel },$$

with *i*_**k**,**m**_ defined analogously. The node conservation principle leads to the condition ${\sum }_{{C}_{k}}({s}_{{\bf{k}},{\bf{m}}}+{i}_{{\bf{k}},{\bf{m}}})=1$, which is to say that the sum of all densities *s*_**k**,**m**_ and *i*_**k**,**m**_ with underlying degree *k*, is one. We then have

8 $${\rho }_{k}=1-\sum _{{C}_{k}}{s}_{{\bf{k}},{\bf{m}}}$$

and

9 $$\rho =\sum _{k}p(k){\rho }_{k},$$

with  *ρ*_*k*_ giving the probability that a randomly selected node with underlying degree *k* will be infected, and *ρ* the probability that any randomly selected node will be infected.

As discussed in the main text, **s** is the ∣*C*∣-dimensional vector storing the densities *s*_**k**,**m**_. In practice, we use lexicographic ordering of the tuples in *C* to define a one-to-one mapping (**k**, **m**) ↦ *i*, for some *i* in the range 1 ≤ *i* ≤ ∣*C*∣, to define the *i*th element of **s**. In Supplementary Note 1, we argue that for fixed *n* and limiting *k* the size of configuration space behaves like *Θ*(*k*^2*n*^). We demonstrate this analytically, and validate it numerically by constructing *C* over a wide range of parameters.

### Master equation transition rates

We indicate in Table 2 the relationship between (**k**, **m**) and its neighbouring classes in configuration space. Classes that can be reached from (**k**, **m**) via ego, neighbour, and edge transitions are shown in the left column, with the corresponding transition type given in the right column. The rates at which nodes flow between classes are given in the middle column. The following notation is used to describe relative changes to the class (**k**, **m**). First, **e**_*j*_ is the *j*th unit vector, and determines the change to a degree vector as a result of a neighbour transition. Second, we define ${\Delta }_{j}^{\pm }=-{{\bf{e}}}_{j}+{{\bf{e}}}_{j\pm 1}$, which determines the change to a degree vector as a result of an edge transition. When an edge increments or decrements state, adjacent nodes lose a *j*-type edge, and gain a *j* ± 1-type edge, all while preserving the underlying degree *k*. Transitions appear as directed edges in the lattice diagram illustrations of configuration space (see Supplementary Note 1).

**Table 2:** Class transition rates.

Configuration	Transition rate	Transition type
$({\bf{k}},{\bf{m}})$	*F*_**k**,**m**_	Ego
${({\bf{k}},{\bf{m}}+{{\bf{e}}}_{j})}$	*β*_*j*_(*k*_*j*_ − *m*_*j*_)	Neighbour
${({\bf{k}},{\bf{m}}-{{\bf{e}}}_{j})}^{* }$	*β*_*j*_(*k*_*j*_ − *m*_*j*_ + 1)	Neighbour
$({\bf{k}}+{\Delta }_{j}^{+},{\bf{m}})$	*μ*_*j*_(*k*_*j*_ − *m*_*j*_)	Positive edge
$({\bf{k}}+{\Delta }_{j}^{+},{\bf{m}}+{\Delta }_{j}^{+})$	*μ*_*j*_*m*_*j*_	Positive edge
${({\bf{k}}-{\Delta }_{j}^{-},{\bf{m}})}^{* }$	*μ*_*j*_(*k*_*j*_ − *m*_*j*_ + 1)	Positive edge
${({\bf{k}}-{\Delta }_{j}^{-},{\bf{m}}-{\Delta }_{j}^{-})}^{* }$	*μ*_*j*_(*m*_*j*_ + 1)	Positive edge
$({\bf{k}}+{\Delta }_{j}^{-},{\bf{m}})$	*ν*_*j*_(*k*_*j*_ − *m*_*j*_)	Negative edge
$({\bf{k}}+{\Delta }_{j}^{-},{\bf{m}}+{\Delta }_{j}^{-})$	*ν*_*j*_*m*_*j*_	Negative edge
${({\bf{k}}-{\Delta }_{j}^{+},{\bf{m}})}^{* }$	*ν*_*j*_(*k*_*j*_ − *m*_*j*_ + 1)	Negative edge
${({\bf{k}}-{\Delta }_{j}^{+},{\bf{m}}-{\Delta }_{j}^{+})}^{* }$	*ν*_*j*_(*m*_*j*_ + 1)	Negative edge

Classes neighbouring (**k**,**m**) in configuration space are shown in the leftmost column. Those marked with and without an asterisk flow into and out of (**k**,**m**), respectively. The rate at which they do so is given in the centre column. Transition types are shown in the rightmost column, and include ego, neighbour and edge transitions. Classes related to (**k**,**m**) by neighbour transitions differ only by **e**_*j*_, the *j*th unit vector. Classes related by edge transitions differ by ${\Delta }_{j}^{\pm }=-{{\bf{e}}}_{j}+{{\bf{e}}}_{j\pm 1}$, that is, by the loss of an edge of type *j*, and the gain of an edge of type *j* ± 1.

Ego transitions occur at rates *F*_**k**,**m**_, and involve the flow of nodes from set *S*_**k**,**m**_ to *I*_**k**,**m**_. As such, no change to the ego’s local neighbourhood (**k**, **m**) takes place, and the transition represents a type of self-edge, or loop, in the lattice representation of configuration space. The rates *F*_**k**,**m**_ are encoded in transmission functions such as those shown in Table 1. Flux measurements of these transitions, such as those in Supplementary Note 7, are expected to be exact. This is because node infection is directly determined by *F*_**k**,**m**_. As nodes are infected at constant rates, we draw the waiting time to infection from an exponential distribution with mean 1/*F*_**k**,**m**_. As such, flux measurements of ego transitions must agree with *F*_**k**,**m**_, by construction. This makes them a useful benchmark for verifying one’s implementation. The rates *F*_**k**,**m**_ are contained in the matrix *W*_ego_.

Neighbour transitions are based on the probability *β*_*j*_*dt* that an uninfected neighbour of an uninfected node becomes infected over an interval *dt*. To calculate *β*_*j*_ we use a straightforward ensemble average over *S*. To obtain the expected fraction of neighbours undergoing transitions, we observe the number of nodes undergoing ego transitions at time *t*, and count the number of neighbour transitions produced as a result. That is, when an uninfected node in class (**k**, **m**) becomes infected, which occurs with probability *F*_**k**,**m**_*dt*, it has *k*_*j*_ − *m*_*j*_ uninfected neighbours that observe this transition, or *k*_*j*_ − *m*_*j*_ nodes undergoing neighbour transitions. The number of such edges across the entire network is given by ∑_*S*_*p*_**k**_(*k*_*j*_ − *m*_*j*_)*F*_**k**,**m**_*s*_**k**,**m**_, where the sum is over all uninfected classes. We compare this with the total number of uninfected-uninfected edges, ∑_*S*_*p*_**k**_(*k*_*j*_ − *m*_*j*_)*s*_**k**,**m**_, giving the neighbour transition rate

10 $${\beta }_{j}dt=\frac{{\sum }_{S}{p}_{{\bf{k}}}({k}_{j}-{m}_{j}){F}_{{\bf{k}},{\bf{m}}}{s}_{{\bf{k}},{\bf{m}}}}{{\sum }_{S}{p}_{{\bf{k}}}({k}_{j}-{m}_{j}){s}_{{\bf{k}},{\bf{m}}}}dt,$$

which has previously been used in master equation solutions of binary-state dynamics on static networks. The rates *β*_*j*_ are contained in the matrix *W*_neigh_, weighted by the values *k*_*j*_ and *m*_*j*_ of the relevant classes (**k**, **m**), as detailed in Supplementary Note 1.

Edge transitions occur at rates *μ*_*j*_ and *ν*_*j*_, and give the probability of edges in state *j* transitioning to state *j* + 1 or *j* − 1, respectively, over an interval *dt*. Their value depends upon the temporal network model in question. In this work, edge transition rates are determined by renewal processes following interevent time distributions *ψ*(*τ*), with complementary cumulative distributions Ψ. If the state of an edge is determined by the number of events *j* having occurred in the preceding time window of duration *η* owing to a renewal process, edge transition rates are

11 $${\mu }_{j}dt=\frac{\Psi * {\psi }^{* j}}{\Psi * {\psi }^{* (j-1)}* \Psi }dt$$

and

12 $${\nu }_{j}dt=\frac{\Psi * {\psi }^{* (j-1)}}{\Psi * {\psi }^{* (j-1)}* \Psi }dt,$$

with

13 $${E}_{j}=\Psi * {\psi }^{* (j-1)}* \Psi$$

giving the probability that a randomly selected edge is in state *j*. It is this quantity that provides the normalising constant for the rates *μ*_*j*_ and *ν*_*j*_. Here, ψ^*j^ is the *j*th convolution power of *ψ*. A complete derivation of these quantities is given in Supplementary Note 1. The Gaver–Stehfest algorithm is used to compute the inverse Laplace transforms, and an efficient numerical procedure reducing *μ*_*j*_ and *ν*_*j*_ to a matrix-vector product is developed in Supplementary Note 5. These expressions hold for *j* > 0, with Eqs. (2) and (3) in the main text giving the special case of *j* = 0 for *E*_*j*_ and *μ*_*j*_, respectively. Regardless of the form of *ψ*, the mean edge state *η*/〈*τ*〉 is always conserved on a network-wide level. Applying Eqs. (11) and (12) at the level of class transitions amounts to a mean field approximation, since flux measurements of Monte Carlo simulation show edge transition rates to deviate slightly from *μ*_*j*_ and *ν*_*j*_ at the class level (**k**, **m**), even if exact for the network as a whole, as shown in Supplementary Note 7.

### Monte Carlo simulation

We simulate networks ${\mathcal{G}}=({\mathcal{V}},{\mathcal{E}})$ composed of a node set ${\mathcal{V}}$ of size *N*, and an underlying edge set ${\mathcal{E}}$. The edge set is produced by a desired degree distribution, wired according to the configuration model. Overlying temporal network activity is initialised to the steady state, such that at time *t* = 0, the time to the first event follows exactly the residual distribution Ψ, in the limit of large networks. Specifically, we set the time to *t* =  −*η*, and draw $| {\mathcal{E}}|$ residual times from Ψ, or one for each edge. Subsequent interevent times are drawn from *ψ*. Advancing in time from  −*η* ensures that a stationary distribution of edge states *E*_*j*_ is achieved exactly at *t* = 0, when we begin to allow node dynamics to evolve. Owing to the large values of interevent time standard deviation studied in this work, out-of-the-box sampling routines were either inefficient or broke down for large *σ*_*τ*_. As such, we develop a simple, yet efficient routine in Supplementary Note 4 based on approximate inverse transform sampling of *ψ* and Ψ, using a bisection method. This is performed on a numerical grid of Ψ values, with relevant details of the probability distributions outlined in detail in Supplementary Note 9. A third-order spline interpolation on a logarithmic scale provides intermediate values of the grid, such that the resultant underlying distribution is close to exact.

Node dynamics are implemented via a Gillespie algorithm, which uses the fact that the waiting time to infection for an uninfected node in class (**k**, **m**) follows an exponential distribution with mean 1/*F*_**k**,**m**_. Initially all nodes are in the uninfected state, and the diffusion process is triggered by low-level background noise at rate *p*. To simulate the temporal network itself, a time-ordered sequence of edge events is implemented in parallel with the node update sequence. This amounts to two separate time-ordered sequences of events executed simultaneously. Algorithms are described in detail in Supplementary Note 4 with pseudocode.

We use the normalised density of noise-induced infections, *ρ*_*f*_, and normalised diffusion time, *t*_*f*_, as measures of the diffusion process. We define these quantities as follows. The probability that a randomly selected node has been infected as a result of external noise is

14 $$\tilde{\rho }(t)=p\int_{0}^{t}(1-\rho (\tau ))d\tau,$$

meaning $0\,<\, \tilde{\rho }\, \le\, \rho$. We define *ρ*_*f*_ as the fraction of infections that are due to noise, ${\rho }_{f}=\tilde{\rho }/\rho$, such that 0 < *ρ*_*f*_ ≤ 1. This value cannot equal zero since there must be at least one noise induced infection, namely, the first infection in the diffusion process. A value approaching *ρ*_*f*_ = 1 means almost all infection is due to external noise. This occurs in the annealed limit, when almost all edges are in state *j* = 0, and network interactions play a vanishingly small role in the diffusion process. As a consequence, the time evolution of the diffusion process is governed by

15 $$\dot{\rho }=p(1-\rho ),$$

whose solution *ρ* = 1 − *e*^−*pt*^ can be inverted to give the time required to the achieve a given density *ρ* of infections relying solely on noise, that is,

16 $$t=\frac{-\mathrm{ln}\,(1-\rho )}{p}.$$

If *t*_*c*_ is the time required in the general case to reach a cutoff density of infections *ρ*_*c*_, normalising *t*_*c*_ by Eq. (16) evaluated at *ρ*_*c*_ defines *t*_*f*_, such that 0 < *t*_*f*_ ≤ 1. A value of *t*_*f*_ = 1 means the system is driven entirely by noise, and a value approaching 0 a rapid diffusion process. An important feature of this work is that *t*_*f*_ and *ρ*_*f*_ seem to be interchangeable, as per the inset of Fig. 2a, and any result shown in terms of *ρ*_*f*_ produces an identical picture in *t*_*f*_.

### Data description

In this work, we use two empirical temporal networks used by ref. ^57^ and references therein, which we describe below. The first is a temporal network of email exchange^57,58^, extracted from the log files of a university email server. The sender, recipient and the timestamp are used to form the network. The data set consists of *N* = 3,188 nodes, and $| {\mathcal{E}}| ={\,}$31,857 underlying edges, such that the average degree is 19.9. A total of 308,730 events were recorded, with a resolution of one second over a period of 81.3 days. An average of 9.69 events occur per edge. We determine the interevent time distribution by taking the subset of edges observing more than one event, of which there are 21,199. The mean interevent time is then calculated to be 〈*τ*〉 = 3.13 days, with standard deviation *σ*_*τ*_ = 6.62 days. This yields a coefficient of variation *σ*_*τ*_/〈*τ*〉 =  2.12.

The second data set is a temporal network of online forum interactions^57,59^. Similar to the email data set, the sender, recipient and the timestamp are extracted from the messages. The data set consists of *N* = 7,083 nodes, and $| {\mathcal{E}}| ={\,}$138,144 underlying edges, such that the average degree is 39.0. A total of 1,428,493 events were recorded, with a resolution of one second over a period of 3,133 days. An average of 10.3 events occur per edge. We determine the interevent time distribution by taking the subset of edges observing more than one event, of which there are 70,902. The mean interevent time is then calculated to be 〈*τ*〉 = 16.6 days, with standard deviation *σ*_*τ*_ = 76.5 days. This yields a coefficient of variation *σ*_*τ*_/〈*τ*〉 = 4.61.

## Supplementary information

Supplementary Information

Peer Review File

## Data Availability

The data that support the findings of this study are openly available through direct request to the authors of original publications.

## Code availability

The code that supports the findings of this study is available upon request from the corresponding authors.
