# Supplementary material for: Dynamics of cascades on burstiness-controlled temporal networks
Source: Nat Commun. 2021 Jan 8;12:133. doi: 10.1038/s41467-020-20398-4 (PMC7794342; doi:10.1038/s41467-020-20398-4)
Supplement: Supplementary file 1 — Supplementary Information [file 41467_2020_20398_MOESM1_ESM.pdf]

Supplementary Information

Dynamics of cascades on burstiness-controlled temporal networks

Samuel Unicomb,[1] Gerardo Iñiguez,[2,3,4] James P. Gleeson,[5] and Márton Karsai[2,1]

[1]*Université de Lyon, ENS de Lyon, INRIA, CNRS, UMR 5668, IXXI, 69364 Lyon, France*
[2]*Department of Network and Data Science, Central European University, A-1100 Vienna, Austria*
[3]*Department of Computer Science, Aalto University School of Science, FI-00076 Aalto, Finland*
[4]*Centro de Ciencias de la Complejidad, Universidad Nacional Autonóma de México, 04510 CDMX, Mexico*
[5]*MACSI and Insight Centre for Data Analytics, University of Limerick, Limerick V94 T9PX, Ireland*

Contents

Supplementary Note 1.	Master equation solution	1
Supplementary Note 2.	Random walk equivalence	6
Supplementary Note 3.	Edge-state distribution	7
Supplementary Note 4.	Monte Carlo simulation	8
Supplementary Note 5.	Laplace transform inversion	10
Supplementary Note 6.	Static network topology	12
Supplementary Note 7.	Mean field approximation	13
Supplementary Note 8.	Skewness and entropy	15
Supplementary Note 9.	Probability distributions	16
References		18

Supplementary Note 1. Master equation solution

In this supplementary note we provide additional information to the master equation solution outlined in the main text. While the configuration space and its transitions was described in the main text, here we discuss a number of binary-state models to which our framework applies. Further, we derive the asymptotic size of configuration space, confirming this result numerically over a wide range of system parameters. Further, we provide lattice diagrams of configuration space and their interpretations. Further, we derive the edge transition rates μ_j and ν_j, along with the distribution of edge states E_j.

Models of binary-state dynamics

In this section we outline how a number of well-known binary-state models may be straightforwardly extended to a temporal setting, and thus be solved using our master equation framework. These models are outlined in Supplementary Table I. Although we have not experimented on recovery models, where infected nodes reenter the uninfected state, it is an effortless extension to our framework. Importantly, the structure of configuration space is unchanged when solving for models that allow for node recovery. If infection involves transitions from set $S_{\boldsymbol{k},\boldsymbol{m}}$ to $I_{\boldsymbol{k},\boldsymbol{m}}$ at rate $F_{\boldsymbol{k},\boldsymbol{m}}$, recovery involves transitions in the opposite direction at rate $R_{\boldsymbol{k},\boldsymbol{m}}$.

Susceptible-Infected-Susceptible model. A straightforward extension to the SI epidemic model is to allow infected nodes to recover the uninfected, or susceptible state, thus defining the SIS model. Recovery takes place at rate δ here. In this way, recovery is independent of local edge dynamics $\boldsymbol{k}$ and $\boldsymbol{m}$. Like the SI model, infection occurs at rate $\boldsymbol{\lambda}\cdot\boldsymbol{m}$, where interaction events on infected edges each increment the transmission rate by λ, meaning bursts on infected edges increase the risk of infection.

Voter model. Here, a node in the uninfected state perceives influence totalling $\boldsymbol{m}\cdot\boldsymbol{w}$ from infected neighbours, and $\boldsymbol{k}\cdot\boldsymbol{w}$ from all neighbours combined. An uninfected node becomes infected with probability $\boldsymbol{m}\cdot\boldsymbol{w}/\boldsymbol{k}\cdot\boldsymbol{w}$. This fraction must lie between zero and one, since even in the presence of arbitrary temporal fluctuations, $\boldsymbol{m}\cdot\boldsymbol{w}$ is clearly bound by $\boldsymbol{k}\cdot\boldsymbol{w}$. As such the extension to temporal networks is natural. A node in the infected state recovers with probability $1-\boldsymbol{m}\cdot\boldsymbol{w}/\boldsymbol{k}\cdot\boldsymbol{w}$, meaning probabilities sum to one. A burst of activity on an infected edge increases the likelihood of infection, while a burst of activity on an uninfected edge lowers the likelihood of infection. This is in contrast with above models where $\boldsymbol{k}$ does not appear in the infection rate.

Language model. In this model, the two states represent the primary language choice of an individual, and the probability of transitioning between states is proportional to the fraction of influence in the neighbourhood, $\boldsymbol{m}\cdot\boldsymbol{w}/\boldsymbol{k}\cdot\boldsymbol{w}$, raised to the power of α, multiplied by the status parameter s or $1-s$ of the respective language. $s=0.5$ for the symmetric case of equal-status languages. Setting $\alpha=1$ then recovers the voter model. In a temporal setting, bursts of interactions between a pair of nodes increases the weighting of that speaker, and thus of the probability of retaining or switching languages, of course depending of the language of the interlocutor.

Lattice diagrams

The state space of configurations C over which our master equation framework applies is shown in Supplementary Figs. (1) and (2), for illustrative values of k and n. The space forms a regular lattice structure, with points in this space representing classes $(\boldsymbol{k},\boldsymbol{m})$, and links between them the allowed transitions in a binary-state model of node dynamics. Points are labelled by their $\boldsymbol{m}$ vector, with the corresponding $\boldsymbol{k}$ vector on the bottom left of the lattice. The $m=\sum_j m_j$ is shown at the top left of the lattice.

A given class is directly connected to those that can be reached via neighbour and edge transitions. In the case of neighbour transitions, $\boldsymbol{k}$ is preserved, and $\boldsymbol{m}$ differs by $\pm\boldsymbol{e}_j$. This corresponds to right diagonal transitions in the lattice diagram, black edges, which are directed in a non-recovery dynamics. The number of infected neigh-

Supplementary Table I. **Additional binary-state models.** A selection of models, labelled on the left, to which our framework can be straightforwardly applied. Parameters introduced by each model are briefly described in the supplementary note. Our framework allows for recovery dynamics, which occurs at rate $R_{\boldsymbol{k},\boldsymbol{m}}$, with infection at rate $F_{\boldsymbol{k},\boldsymbol{m}}$.

model	$F_{\boldsymbol{k},\boldsymbol{m}}$	$R_{\boldsymbol{k},\boldsymbol{m}}$
SIS	$\boldsymbol{\lambda}\cdot\boldsymbol{m}$	δ
voter	$\boldsymbol{m}\cdot\boldsymbol{w}/\boldsymbol{k}\cdot\boldsymbol{w}$	$1-\boldsymbol{m}\cdot\boldsymbol{w}/\boldsymbol{k}\cdot\boldsymbol{w}$
language	$s(\boldsymbol{m}\cdot\boldsymbol{w}/\boldsymbol{k}\cdot\boldsymbol{w})^\alpha$	$(1-s)(1-\boldsymbol{m}\cdot\boldsymbol{w}/\boldsymbol{k}\cdot\boldsymbol{w})^\alpha$

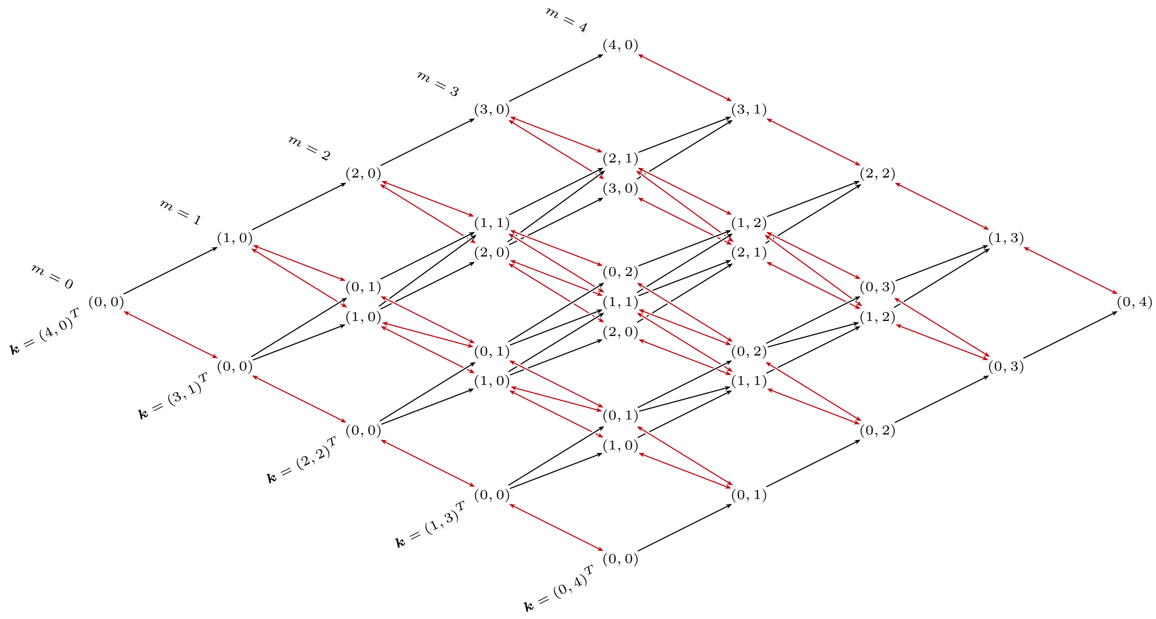

Supplementary Figure 1. **Lattice diagram of temporal network configuration space.** Temporal network configuration space for a node of total degree $k = 4$, with $n = 2$ allowed edge states. Nodes in this lattice are labelled by their infected degree vector $\boldsymbol{m}^T$, with corresponding $\boldsymbol{k}$ shown bottom left. Right-diagonal transitions indicate neighbour infection, and left-diagonals the increments and decrements between edge-states $j = 0$ and 1 that may occur in models of temporal networks. Right-diagonal transitions preserve the degree vector $\boldsymbol{k}$, indicated at the bottom of these diagonals. Left-diagonal transitions preserve total infected neighbour count m, shown at the top of these diagonals.

bours m is incremented by one after these transitions. In the case of edge transitions, neighbouring $(\boldsymbol{k}, \boldsymbol{m})$ classes differ by $\Delta_j^{\pm} = -\boldsymbol{e}_j + \boldsymbol{e}_{j\pm1}$, meaning they lose an edge of type j, and gain an edge of type $j+1$ or $j-1$. These transitions are the red left-diagonal edges in Supplementary Figs. (1) and (2), and are bidirectional. The total number of infected neighbours m is preserved during these transitions.

Supplementary Fig. 2 illustrates that the number of points in configuration space does not change when solving over temporal networks relative to edge heterogeneous static networks. For instance, a temporal model where edge state is bound by $n = 1$ has a C that is no different to that of a two layer multiplex, if the underlying degree distribution is the same. Crucially it is the number of transitions that differ, expressed in the $W_{ego} + W_{neigh} + W_{edge}$ formulation of the master equation.

Size of configuration space

The most important quantity in determining the feasibility of a master equation solution is the number of points in its configuration space C. It is this quantity that determines the size of the resultant system of differential equations, with each point associated to a rate equation for $s_{\boldsymbol{k},\boldsymbol{m}}$. In this section, we estimate analytically the asymptotic growth rate of C, which we then validate using extensive numerical construction.

First, we note that configuration space is finite if and only if the degree distribution has some cutoff, which we call k here, and if the number of allowed edge states n is finite. If these conditions are satisfied, we can approximate the size of C analytically, as shown in Supplementary Fig. 3. We shall be interested in the case where n is fixed, and k is arbitrarily large. Our approach is to first calculate the growth rate of C_k, the number of configurations with total degree k. This itself entails $|C_{\boldsymbol{k}}|$, the number of configurations with degree vector $\boldsymbol{k}$. Once these quantities are known, it is straightforward to estimate the total size of a system whose degree distribution runs from 0 to a cutoff k.

The size of C_k can be written exactly as

$$|C_k| = \sum_{\boldsymbol{k}} \prod_{j=1}^{n} (k_j + 1), \tag{1}$$

where the sum runs over all degree vectors $\boldsymbol{k}$ with total degree k, and the summand represents $|C_{\boldsymbol{k}}|$, which amounts to the number of partial degree vectors $\boldsymbol{m}$ corresponding to a given $\boldsymbol{k}$. To see this, note that $C_{\boldsymbol{k}}$ itself is composed of all configurations where $0 \leq m_j \leq k_j$ is satisfied, for all j, for a given $\boldsymbol{k}$. Then, the k_j neighbours of a node that are in state j have $k_j + 1$ possible inactive, active configurations, given by $m_j = 0, \ldots, k_j$.

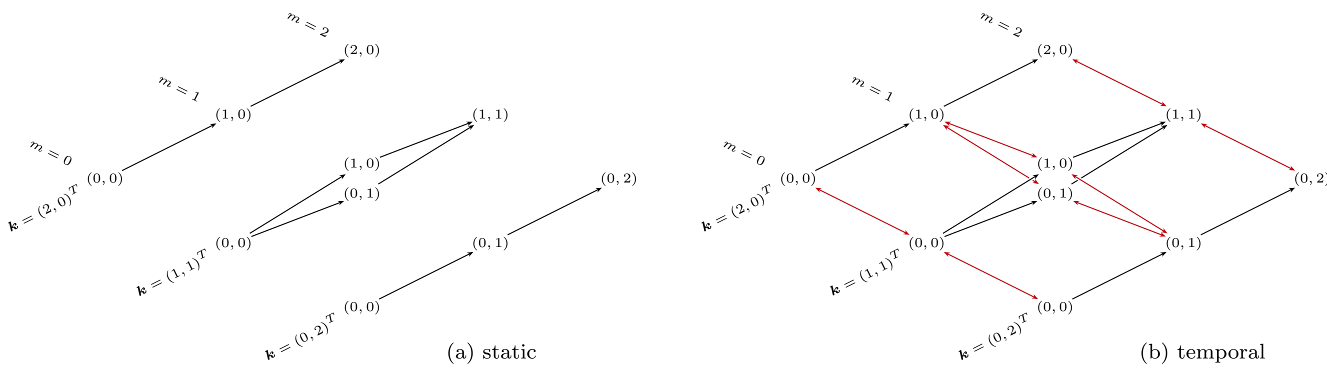

Supplementary Figure 2. **Comparison of static and temporal configuration space.** Allowed transitions in static networks, (a), and temporal networks, (b), for a $k = 2$ degree node with an $n = 2$ level edge-state set. In contrast to static networks, temporal networks allow what we call edge transitions, left-diagonals in (b). The static case may occur in a bimodal weighted network, or a two-layer multiplex network. See caption in Supplementary Fig. 1 for interpretation. Note that the number of configurations $(\boldsymbol{k}, \boldsymbol{m})$, or the number of rate equations in the master equation, is identical in each case.

Since each k_j value is independent, the total number of configurations in $C_{\boldsymbol{k}}$ is given by the product over the n edge types.

To obtain the growth rate of Supplementary Eq. (1), we estimate the value of two quantities. The first is the number of terms in the sum over $\boldsymbol{k}$, and the second the expected value of its summand, $C_{\boldsymbol{k}}$. Since the sum is over all degree vectors $\boldsymbol{k}$ whose elements add to k, it can be enumerated by counting the number of ways of placing k objects into n containers. This is equivalent to choosing k from $k + n - 1$. Such a binomial grows as $\Theta(k^{n-1})$. Second, to determine the expected size of $C_{\boldsymbol{k}}$ for a given k, it is clear that for any degree vector $\boldsymbol{k}$, the number of corresponding $\boldsymbol{m}$ is bounded by $(k+1)^n$, since we clearly have $k_j \leq k$ for all j, with equality holding only when $n = 1$. Although technically this only provides an upper bound, we propose that it is actually stricter, growing as $\Theta(k^n)$.

Given that the number of terms in Supplementary Eq. (1) grows like $\Theta(k^{n-1})$, and that the mean value of its summand arguably grows like $\Theta(k^n)$, we estimate that the number of configurations with total degree k grows like

$$|C_k| = \Theta(k^{n-1}) \times \Theta(k^n) = \Theta(k^{2n-1}). \tag{2}$$

Although we have not included such a figure, this result can be validated in the manner of Supplementary Fig. 9. That is, we construct the set C_k numerically, iterating through all $\boldsymbol{k}$ satisfying k, and all $\boldsymbol{m}$ satisfying that $\boldsymbol{k}$, before plotting its actual size alongside its estimated asymptotic value. In this way, we can confirm that C_k indeed grows like k^{2n-1}. Incidentally, this represents the complete configuration space for a k-regular network.

Finally, we estimate the total number of points in C, assuming that the degree distribution runs from zero to the cutoff k. To do this, we observe that the number of $\boldsymbol{k}$ for each k increases monotonically, and does so gradually when k is large. As such, there will be $k + 1$ degrees to sum over, allowing us to write

$$|C| = \Theta(k) \times \Theta(k^{2n-1}) = \Theta(k^{2n}) \tag{3}$$

for the total configurations $(\boldsymbol{k}, \boldsymbol{m})$ in C. Although far from rigorous, our derivation appears to be validated by Supplementary Fig. 3, where we observe close agreement between the asymptotic term, and brute force enumeration of numerically constructed C. Note that as we display Supplementary Fig. 3 on a log-log scale, polynomials appear as straight lines whose slope is given by their leading order, $2n$, and whose vertical intercept is given by the multiplicative constants associated with Θ notation. These coefficients are obtained by hand, choosing the intercept such that the dashed line fits the data, or solid line.

A consequence of these results is that a precise master equation implementation of real systems is quite impractical. Real systems often include hubs, or nodes whose degrees reach into the tens of thousands. In the presence of edge heterogeneity, we have seen that such systems are prohibitively large. For example, a four-level multiplex with a maximum total degree of $k = 10^4$ is out of reach computationally, as it contains 10^{27} terms, as per Supplementary Fig. 3. Nevertheless, a great deal of intuition can be built be studying the behaviour of more tractable systems, by imposing cutoffs on the maximum degree, and formulating the system in terms of low edge heterogeneity n, as we have done in this work.

Calculating μ_j and ν_j for renewal processes

In this section, we calculate the rates of positive and negative edge transitions μ_j and ν_j in the stochastic temporal network model discussed in the previous section.

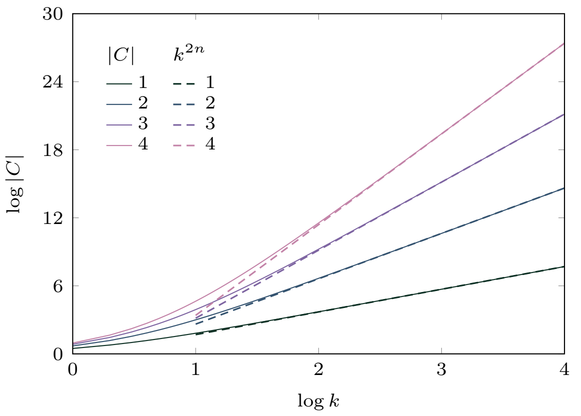

Supplementary Figure 3. **Asymptotic size of configuration space.** Solid curves are $|C|$, the actual size of configuration space C, found by direct numerical enumeration. Dashed curves give the asymptotic size, $\Theta(k^{2n})$, show for $n = 1, 2, 3$ and 4. In this figure k represents the cutoff degree, with C assumed to be composed of nodes ranging from total degree 0 to k. The slopes of the dashed and solid lines are in excellent agreement.

Here, $\mu_j dt$ gives the probability that at time t, for a renewal process having already produced j events in the preceding time window of duration η, a $(j+1)$-th event is observed between time t and $t + dt$. Conversely, $\nu_j dt$ gives the probability of an event exiting the η window. This is illustrated in Supplementary Fig. 4. At the outset, describing such a process with constant rates μ_j and ν_j seems inappropriate, as it is memoryless only in so-called event space. In this representation, a renewal process is nothing other than a sequence of trials, $\{\tau_1, \tau_2, \ldots\}$, or the random sampling of a value τ from a distribution $\psi(\tau)$. In this sense, the process is memoryless. However, in the resulting time-series for continuous t, the process is non-Markovian, as the time of the next event was decided at the time of the previous event, and the probability of an event occurring at a time between these points is zero. This is in contrast to a Poisson process, where the change in edge state due to the occurrence of an event is constant in time. Thus, on a microscopic level, where we observe a stochastic process on a single edge, a rate description is nonsensical.

We note, however, that in our master equation formalism, classes $(\boldsymbol{k}, \boldsymbol{m})$ really represent ensembles of nodes, and although constant rates cannot be identified on a microscopic level, useful quantities do exist on a network-wide macroscopic level. That is, calculating the fraction of j-type edges that change state over an interval dt turns out to be strongly heterogeneous for varying j, which is clearly not the case for a Poisson process. The heterogeneity of the distribution ψ is reflected in the heterogeneity of μ, ν and E.

The rate of positive edge transition $\mu_j dt$ is calculated by finding the probability of a $(j+1)$-th event occurring over a given interval dt, on the condition that j events have already been produced in the preceding time interval of duration η. This is illustrated in Supplementary Fig. 4(a), where we set $t = \eta$ for convenience. In Supplementary Fig. 4, the interevent times $\tau_1, \ldots, \tau_j$ are drawn from the distribution ψ, and the times τ' and τ'' from its complementary cumulative distribution Ψ, also known as the residual time distribution. It is defined as

$$\Psi(\tau) = \int_{\tau}^{\infty} \psi(t) dt, \tag{4}$$

and gives the probability that the time between events is of duration at least τ. We introduce the domain T of times spanned by the configurations allowed in Supplementary Fig. 4, or the times $t_1 < t_2 < ... < t_j$ in an interval of duration η, such that $t_j - t_1 < \eta$ and $t_{j+1} - t_1 < \eta$. Note that consecutive event times t_j and t_{j+1} cannot coincide, with interevent times drawn from distributions ψ and Ψ, which defined over positive τ. We write

$$\begin{aligned} T = (0, \eta] \times (0, \eta - t_1] \times (0, \eta - t_2] \times \ldots \\ \ldots \times (0, \eta - t_{j-2}] \times (0, \eta - t_{j-1}] \subseteq \boldsymbol{R}_+^j, \end{aligned} \tag{5}$$

where $\boldsymbol{R}_+^j$ is the j-dimensional space of positive real numbers. The probability of observing the configuration in Supplementary Fig. 4(a) is $\Psi(\tau')\psi(\tau_1)\ldots\psi(\tau_j)$, which is the same as $\Psi(t_1)\psi(t_2 - t_1)\ldots\psi(\eta - t_j)$ given that we've set the time t to η for simplicity. Similarly, the configuration in Supplementary Fig. 4(b) is observed with probability $\Psi(\tau')\psi(\tau_1)\ldots\psi(\tau_{j-1})\Psi(\tau'')$, which is the same as $\Psi(t_1)\psi(t_2 - t_1)\ldots\psi(t_j - t_{j-1})\Psi(\eta - t_j)$. The weighted sum of all such configurations yields the probability of observing a j type edge undergoing a transition to state $j+1$ over an interval dt, and the probability of randomly selecting an edge in state j, as shown in Supplementary Fig. 4(a) and (b), respectively. The only difference is the final term, which is drawn either form ψ or Ψ. With respect to the domain T, these sums can be written

$$\begin{aligned} \int_T dt_1 \ldots dt_j \Psi(t_1)\psi(t_2 - t_1)\psi(t_3 - t_2) \times \ldots \\ \ldots \times \psi(t_j - t_{j-1})\psi(\eta - t_j) = \Psi * \psi^{*j}, \end{aligned} \tag{6}$$

and

$$\begin{aligned} \int_T dt_1 \ldots dt_j \Psi(t_1)\psi(t_2 - t_1)\psi(t_3 - t_2) \times \ldots \\ \ldots \times \psi(t_j - t_{j-1})\Psi(\eta - t_j) = \Psi * \psi^{*(j-1)} * \Psi, \end{aligned} \tag{7}$$

respectively. Here, ψ^{*j} is the j-th convolution power of ψ, and is discussed at length in following sections. To obtain the rate μ_j at which edges in state j transition to state $j+1$, Supplementary Eq. (6) must be normalised by Supplementary Eq. (7), the probability E_j that a randomly selected edge is in state j. Schematically, this corresponds to normalising the transition in Supplementary Fig. 4(a) by those in Supplementary Fig. 4(b). Note that

$\nu_j dt$, the probability that an edge in state j forgets an event over an interval dt is the same as μ_{j-1} under time reversal, up to the normalising constant. As such, the rates μ_j and ν_j, along with the distribution E_j, can be written compactly as

$$\mu_j dt = \frac{\Psi * \psi^{*j}}{\Psi * \psi^{*(j-1)} * \Psi} dt \tag{8}$$

and

$$\nu_j dt = \frac{\Psi * \psi^{*(j-1)}}{\Psi * \psi^{*(j-1)} * \Psi} dt, \tag{9}$$

with

$$E_j = \Psi * \psi^{*(j-1)} * \Psi. \tag{10}$$

These quantities can be calculated either numerically or analytically, depending on the tractability of the chosen distribution ψ. In general, if $\psi(\tau)$ is locally integrable, then the Laplace transform of ψ and Ψ exists and allows us to calculate the convolution as a product in the frequency domain, which will be useful especially if j is large. Since we don't impose any cutoffs on ψ and Ψ in the text, j indeed can grow arbitrarily large, under bursty dynamics. If we denote the Laplace transform of $\psi(\tau)$ by

$$\mathcal{L}\left\{\psi(\tau)\right\} = \hat{\psi}(s) = \int_0^\infty \psi(\tau) e^{-s\tau} d\tau \tag{11}$$

then the transform of Supplementary Eq. (4) can be written as

$$\hat{\Psi}(s) = \frac{1-\hat{\psi}(s)}{s}. \tag{12}$$

Finally, we can argue that by induction from $j = 0$, and using the fact that the distribution E is constant at stationarity of the renewal process, that $\mu_j E_j = \nu_{j+1} E_{j+1}$, meaning that along with E_j, the rates μ_j and ν_j are stationary. The observed experimental rates μ_j, ν_j and E_j match exactly the predicted values, for increasingly large networks.

Illustration using the exponential distribution

In this section we explicitly calculate the edge transition rates μ_j and ν_j for an exponential interevent time distribution ψ. This is an exercise to illustrate the Laplace inversion procedure, in general we calculate these quantities numerically, as described in Supplementary Note 5. The exponential distribution is an important benchmark in this work, and we consider two alternative generalisations, namely the gamma and Weibull distributions. Both reduce to the exponential distribution when $\sigma_\tau = \langle\tau\rangle = 1$, and recover the rates given here. Consider

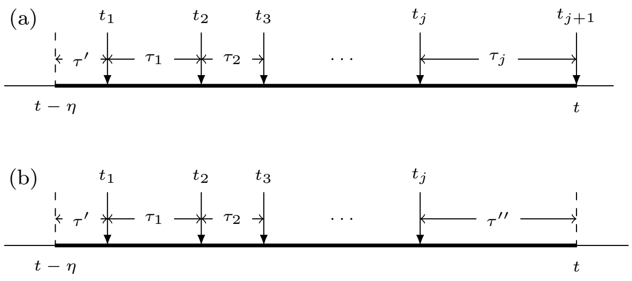

Supplementary Figure 4. **Enumeration of edge state configurations.** Configuration of j events occurring over an interval of length η, where η amounts to memory of observed events. In plot (a) we enumerate all configurations of edges in state j at time t, with a $(j+1)$-th event occurring over the interval $t < t_{j+1} < t + dt$. In plot (b) we enumerate all the configurations of edges in state j. We use this to calculate the rate of positive edge transition μ_j, and edge state distribution E_j, respectively. Interevent times $\tau_1, \ldots, \tau_j$ are drawn from ψ, whereas τ' and τ'' are drawn from Ψ, defined in the text.

then the exponential distribution with mean $\langle\tau\rangle$ defined by

$$\psi(\tau) = \frac{1}{\langle\tau\rangle} e^{-\tau/\langle\tau\rangle} \tag{13}$$

with

$$\Psi(\tau) = e^{-\tau/\langle\tau\rangle}, \tag{14}$$

having transforms

$$\hat{\psi}(s) = \frac{1}{\langle\tau\rangle s + 1} \tag{15}$$

and

$$\hat{\Psi}(s) = \frac{1-\hat{\psi}}{s} = \frac{\langle\tau\rangle}{\langle\tau\rangle s + 1}, \tag{16}$$

respectively. Substituting these transforms into Supplementary Eq. (10), and applying the convolution theorem allows us to calculate the expected size of the set of edges in state j, which is also the normalising constant in the rates μ_j and ν_j, as the expression for $\mathcal{L}\left\{E_j\right\}$ simplifies to to

$$\hat{\Psi} \cdot \hat{\psi}^{j-1} \cdot \hat{\Psi} = \frac{\langle\tau\rangle^2}{\left(\langle\tau\rangle s + 1\right)^{j+1}}. \tag{17}$$

We use the fact that $\mathcal{L}^{-1}\{\frac{1}{s^{j+1}}\} = \eta^j/j!$, for integer j, a known Laplace transform relating to the gamma function. We use also the translation property $\mathcal{L}^{-1}\{\hat{\psi}(s + a)\} = e^{-a}\psi$, which directly results from the definition Supplementary Eq. (11). The inverse Laplace transform of the above expression can then be written explicitly as

$$\Psi * \psi^{*(j-1)} * \Psi = \langle\tau\rangle \frac{e^{-\eta/\langle\tau\rangle}}{j!}\left(\frac{\eta}{\langle\tau\rangle}\right)^j, \quad (18)$$

meaning E is simply the Poisson distribution with mean $\eta/\langle\tau\rangle$, up to a factor of $\langle\tau\rangle$. This is expected, due to our construction of the memory window, and the fact that an exponential interevent time distribution recovers a Poisson process. Similarly, the Laplace transform of the numerator in Supplementary Eqs. (8) and (9) can be used to calculate

$$\Psi * \psi^{*j} = \frac{e^{-\eta/\langle\tau\rangle}}{j!}\left(\frac{\eta}{\langle\tau\rangle}\right)^j \quad (19)$$

and

$$\Psi * \psi^{*(j-1)} = \frac{e^{-\eta/\langle\tau\rangle}}{(j-1)!}\left(\frac{\eta}{\langle\tau\rangle}\right)^{j-1}, \quad (20)$$

yielding

$$\mu_j dt = \frac{1}{\langle\tau\rangle} dt \quad (21)$$

and

$$\nu_j dt = \frac{j}{\eta} dt, \quad (22)$$

after normalising by $\Psi * \psi^{*(j-1)} * \Psi$. In this special case of exponentially distributed τ, we are able to derive these rates using much simpler arguments, namely with the definition of the Poisson process, and the Poisson distribution. Crucially, μ_j has no j dependence here, which clearly expresses the memoryless property of the Poisson process. These rates may be verified by simulating an ensemble of independent, stationary renewal processes, and observing the flux in the system over an interval Δt. The flow through the set E_j over that interval, scaled by the size of that set and the size of the measurement window, give the rates μ_j and ν_j. Alternatively, by simulating a single renewal process for a sufficiently long time, and measuring its change in behaviour over each interval Δt, one obtains rates μ_j and ν_j that are identical to those calculated in the ensemble.

Convolution powers, an aside

In order to calculate the distribution of edge states E_j, as well as the mean field edge transition rates μ_j and ν_j, we need an efficient method for computing convolution powers. In general a convolution is defined for two real valued functions f and g over the domain $f, g : (-\infty, \infty) \to \mathbb{R}$. However, in the case where f and g take non-negative values, as is the case in our study, the convolution reduces to

$$(f * g)(t) = \int_{-\infty}^{\infty} f(\tau) g(t-\tau) d\tau = \int_0^t f(\tau) g(t-\tau) d\tau. \quad (23)$$

We use this to express edge-state properties, Supplementary Eqs. (6) and (7), as convolutions over the non-negative reals. It is worthwhile noting the convention that if ψ^{*j} is the j-th convolution power, or

$$\psi^{*j} = \underbrace{\psi * \psi * \ldots * \psi}_{j \text{ terms}}, \quad (24)$$

then the zeroth order convolution, $\psi^{*0} = \delta_0$, is simply the Dirac delta function, the identity of convolution. This is used in the $j = 1$ case of Eqs. 8, 9 and 10.

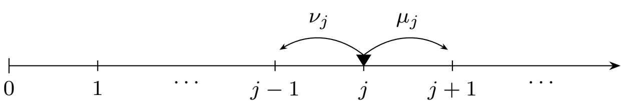

Supplementary Figure 5. **Random walk interpretation of edge state.** Positive and negative edge transition rates, μ_j and ν_j, act as a signature of the non-Markovianity in the renewal process model. On a macroscopic level, this model is indistinguishable from a random walk as illustrated above, where the transition rates are provided by μ_j and ν_j by construction. In contrast, this equivalence is broken when taking into account node dynamics on the level of classes $(\boldsymbol{k}, \boldsymbol{m})$, as we shown in Supplementary Fig. 10. See also Supplementary Fig. 6 for illustrative values of the rates μ_j and ν_j for varying σ_τ.

Supplementary Note 2. Random walk equivalence

In this Supplementary Note, we discuss the expected steady-state network topology that emerges due to our stochastic temporal model. In particular, we describe the existence of a pure Markovian system with identical macroscopic dynamics to our non-Markovian renewal process model. We consider the dependence of the edge transition rates μ_j and ν_j on our choice of interevent time distribution ψ, and in particular, its parameterisation in terms of standard deviation σ_τ.

We assume an arbitrarily large network consisting of independent, stationary renewal processes, such that the time to the next event at any time t follows the residual distribution $\Psi(\tau)$. We illustrate in Supplementary Fig. 6 the edge transitions rates μ_j and ν_j that emerge from a gamma interevent time distribution $\psi(\tau)$, for increasing values of standard deviation σ_τ. Since μ_j and ν_j are heterogeneous, they can be interpreted as providing a signature of the non-Markovianity inherent to the renewal process microscopically. For instance, when $\sigma_\tau = \langle\tau\rangle = 1$, the gamma distribution reduces to an exponential distribution, corresponding to a Poisson pro-

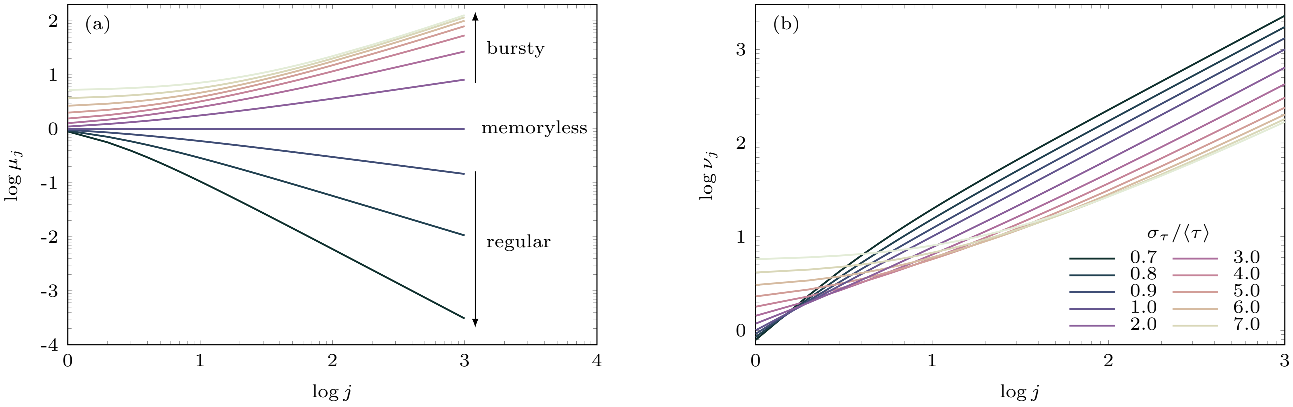

Supplementary Figure 6. **Illustration of edge transition rates, or signatures of non-Markovianity.** Positive and negative edge transition rates, μ_j and ν_j, respectively, for a gamma distributed interevent time with mean $\langle\tau\rangle = 1$. On a macroscopic scale, these values act as a signature of the implicit memory dependence characterised by $\psi(\tau)$ and η. On this scale, our renewal process model is indistinguishable from a random walk model of edge state, where the state j increments and decrements at rates μ_j and ν_j. Legend in (b) applies also to (a). Observer memory and mean interevent time are given by $\eta = \langle\tau\rangle = 1$, according to our stochastic temporal network model. The exponential distribution is recovered in the case of $\sigma_\tau = \langle\tau\rangle = 1$, producing a memoryless system, as indicated.

cess. That this process is memoryless is reflected in the edge transition rate $\mu_j = 1/\langle\tau\rangle$, being homogeneous in j. The greater the departure from the Poisson process, the greater the heterogeneity in j, illustrated in Supplementary Fig. 6(a).

A central result in this work is to note that for an uncorrelated ensemble of edges, the renewal process model is indistinguishable from a continuous-time Markov chain, namely, a one-dimensional biased random walk, as illustrated in Supplementary Fig. 5. In other words, the probability of a randomly selected edge undergoing a transition over an interval dt in the renewal process model is trivially identical to a Markov chain where by construction, transitions occur at rates μ_j and ν_j. Indeed, since all temporal network information is stored in μ_j, ν_j and E_j in the master equation, any class of system producing a given set of μ_j, ν_j and E_j values has the same predicted dynamics. Since in the special case of a Markov chain transition rates are exact at all scales (both locally on the scale of a single edge, and globally on the scale of an uncorrelated ensemble), the master equation solution is exact here. Since Supplementary Eqs. (8) and (9) represent a mean field approximation on the scale of classes $(\boldsymbol{k}, \boldsymbol{m})$ in the renewal process model, deviations in the master equation solution emerge here. These errors provide a measure of the extent to which non-Markovian dynamics can be captured by the heterogenity of a simple Markov chain.

Supplementary Note 3. Edge-state distribution

In this Supplementary Note we mention some basic properties of the edge state distribution. First, for all choices of $\psi(\tau)$ and η in this work, a useful conserved quantity is the expected edge state, or the expected number of events per edge across the entire network. If $\langle\tau\rangle$ is the mean of this distribution, and η is observer memory, the expected edge state is $\langle E\rangle = \eta/\langle\tau\rangle$, and is useful for monitoring the accuracy of the implementation. Further, note that if $\mathcal{E}$ is the set of underlying edges in the network, the superposition of $|\mathcal{E}|$ renewal processes converges to a exponential distribution with mean $\langle\tau\rangle/|\mathcal{E}|$, for large $\mathcal{E}$. As such, we expect $|\mathcal{E}|dt/\langle\tau\rangle$ events per time window dt in simulation.

Temporal network percolation transition

The probability that a randomly selected edge is in state zero is given by ξ_E. One can use ξ_E to determine whether active edges in the network, the set of all edges in state one or higher, are expected to form a giant component at any given time. If a giant component exists, we say that percolation has taken place. If percolation does not occur, nodes form finite active clusters whose size goes to zero in the limit of large networks. This is true even if the underlying network itself consists of a giant component. The percolation transition helps to explain sharp transitions in diffusion dynamics, separating regimes of fast and slow information diffusion. The percolation condition for a configuration model network with degree distribution $q(k)$ is given by

$$\sum_{k=0}^{\infty} k(k-2)q(k) = 0, \tag{25}$$

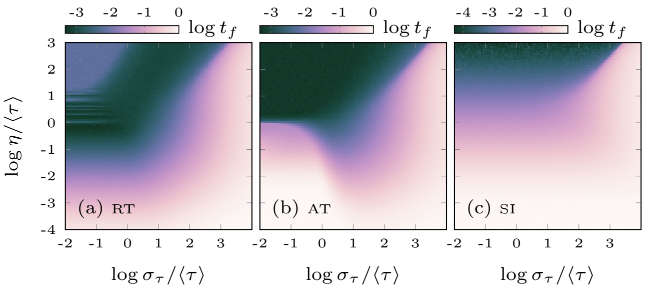

Supplementary Figure 7. **Heat map comparison of spreading models.** Spreading time t_f for the relative and absolute threshold models, (a) and (b) respectively, and SI model, (c). Interevent time distribution is Weibull, and the underlying network is lognormal with mean $\langle k\rangle = 7$ and standard deviation $\sigma_k = 0.5$. The temporal network phase transition is in evidence in the top right of each plot.

where

$$q(k) = \sum_{l\geq k}^{\infty} p(l)\binom{l}{k}(1-\xi_E)^k \xi_E^{l-k}, \tag{26}$$

is the degree distribution obtained by randomly removing a fraction ξ_E of edges from a network with degree distribution $p(k)$, which is effectively the network induced when removing edges in state zero in out temporal model. The percolation transition is responsible for a corresponding transition in diffusion dynamics, echoed in Supplementary Fig. 7.

Maximum edges state

In our analytic solution, we introduce n to denote the maximum edge state j. In experiment, no such restrictions are imposed when sampling the interevent time distributions ψ and Ψ, in contrast to related work [1] where it is common to introduces upper lower bounds on τ. As a consequence, bursts in activity can lead to arbitrarily large edge states j. However, as we see in the structure of configuration space, if n is the maximum edge state allowed in the system, the number of equations grows like $\Theta(k^{2n})$. Clearly, in the interest of the numerical implementation of the master equation solution, n cannot be arbitrarily large.

It is noteworthy that despite edge state being unrestricted in simulation, the resultant diffusion dynamics can be accurately solved using relatively small values of n. Consider that large bursts are most common when the interevent time standard deviation σ_τ is large. In this regime, the fraction of edges in state $j = 0$ is significant. As a consequence, nodes observing bursts of activity on some edges frequently observe no activity on others. Indeed for large enough σ_τ, it is rare for a node to observe more than on active neighbour, with that active neighbours generally being in a very large state j. For the relative threshold (RT) model, such a node is infected with high probability if the neighbouring node is active, since the threshold ϕ is guaranteed to be overcome here. Similarly, for the absolute threshold (AT) model, any infected neighbour in state $j = \lceil M_\phi \rceil$ or higher is likely to adopt. Finally, for the susceptible-infected (SI) model, consider that the duration of a spike in activity is on the order of η, i.e., the duration of observer memory. Since the infection rate increases proportionally to the size of the burst, a large burst leads to infection soon after its observation, and long before they have left the time window. Since the local neighbourhood of the newly infected node is likely sparse in this setting, a much smaller burst would lead to an identical diffusion outcome. Effects such as these mean that surprisingly low value of n are sufficient to accurately model the diffusion process.

Supplementary Note 4. Monte Carlo simulation

In this Supplementary Note we discuss the Monte Carlo simulation methods used in this work. Since node dynamics do not feed back into edge dynamics, we assume a steady state renewal process is in place at $t = 0$. This involves initialising the system with one τ drawn from the tail distribution Ψ. We start the simulation at $t = -\eta$, so that at time $t = 0$, the system is at steady state.

Parallel event sequences

Our model of node dynamics is Markovian, since an uninfected node v becomes infected at a constant rate F_v. In the case of threshold models, the transmission rate is a step function, whose upper value is set conventionally to one. A straightforward Monte Carlo simulation in this case is to advance in time with fixed intervals $\Delta t = \frac{1}{N}$, where N is network size, and randomly selecting a node v to trigger with probability F_v at each step. This approach is unsuitable when transmission rates are unbounded, since the time step Δt cannot be rescaled *a priori* to preserve the random node selection approach. This is the case for the SI rule in our model. We define the transmission rate here to be proportional to edge state $F_{\boldsymbol{k},\boldsymbol{m}} = \max(p,\ \boldsymbol{m}\cdot\boldsymbol{\beta})$, where $\boldsymbol{\lambda} = \lambda\boldsymbol{w}$, for a node in class $(\boldsymbol{k},\boldsymbol{m})$. Since $\boldsymbol{m}$ and $\boldsymbol{\lambda}$ are unbounded in our simulations, as we impose no restriction on ψ and Ψ, bursts of activity due to our renewal process model can result in arbitrarily high F.

For this reason, we prefer an event-based Gillespie algorithm, which is equivalent, but advances in time by jumping to the next event, rather than by uniform increments Δt. In the remainder of this section we discuss the implementation of sequences of these events.

Algorithm 1 Static network event sequence

procedure MONTECARLOSTATIC(Ξ_v)
  while Ξ_v not empty **do**
    apply head of Ξ_v
  end while
end procedure

For a graph $\mathcal{G}(\mathcal{V}, \mathcal{E})$, we define two types of events, node events, defined over the node set $\mathcal{V}$, and edge events, defined over the edge set $\mathcal{E}$. A node event is implemented as the tuple $\{t_v, v\}$, and edge events $\{t_e, e_{uv}, s\}$, respectively. Node events amount to the infection of node v, at a time t_v, and edge events the positive or negative change in the state of edge e_{uv}, depending on the sign of the indicator $s = \pm 1$, according to our stochastic temporal network model. Monte Carlo simulation is implemented as two time ordered, dynamic sequences of events, Ξ_v and Ξ_e, for node and edge event types, respectively. While Ξ_e is independent of node dynamics, both node end edge events feed back and cause a potential reordering of Ξ_v, as explained below. The node event sequence is initially of size $N = |\mathcal{V}|$, since we have one event for each node in the network, with the leading event being removed when all preceding edge activity has been carried out, i.e., when $t_v < t_e$, for the leading terms in each sequence. The action carried out is best illustrated by considering a static network, Algorithm 1. Here, the *apply head* instruction means to trigger the node in question, remove it from Ξ_v, and update the transmission rates of its neighbours, and in turn, their position in Ξ_v. In our model, the edge event sequences has approximately constant size, apart from some fluctuations due to the fact that $\eta/\langle\tau\rangle$ is only the expected edge state, the absolute number of events in η-memory can go up and down. As networks increase in size, the size of the edge event sequence Ξ_e converges to $(2 + \eta/\langle\tau\rangle)\,|\mathcal{E}|$. Assuming that a non-zero level of noise is present, $p > 0$, then the Ξ_v will eventually be emptied.

Algorithm 2 Temporal network event sequences

procedure MONTECARLOTEMPORAL(Ξ_v, Ξ_e)
  $t_v, t_e \leftarrow$ dequeue Ξ_v, Ξ_e
  while Ξ_v not empty **do**
    while $t_e < t_v$ **do**
      apply head of Ξ_e
      $t_v, t_e \leftarrow$ dequeue Ξ_v, Ξ_e
    end while
    apply head of Ξ_v
    $t_v \leftarrow$ dequeue Ξ_v
  end while
end procedure

Algorithm 2 is the temporal extension of Algorithm 1. In each, the time of infection of every node v is determined at $t = 0$. This is done by drawing from an exponential distribution with mean F_v, i.e., taking the natural logarithm of a uniform random variable on $(0, 1)$, divided by $-F_v$. A node event is permanently erased from the sequence if $t_v < t_e$, as mentioned. At this time, neighbours u of v have their transmission rates F_u recalculated, as in Algorithm 1, as they now have an additional infected neighbour. Additionally, the infection time of a node is recalculated if a change in its local neighbourhood takes place, such as activity on an adjacent edge, or the infection of a neighbouring node. Since event sequences are time ordered, a node event is first erased from its position in the sequence, and then reinserted when such a calculation takes place. No such reordering take place for the edge event sequence, as once an event is inserted here, it is only removed when its time t_e is at the front of the queue. While $t_e < t_v$, where the events in question are the leading events of each queue, there are two possible actions for the *apply head* instruction for Ξ_e in Algorithm 2. The first, if $s = +1$, the leading edge event is erased and replaced with two new events on the same edge e_{uv}, occurring at time $t_e + \tau$, where τ is drawn from the interevent time distribution ψ. At the same time, an event is inserted for $t_e + \tau + \eta$, with $s = -1$, corresponding to the decrementing of that same edge η time steps later. If an $s = -1$ edge event is leading the sequence, and we still have $t_e < t_v$, then the event is removed from the sequence without replacement.

In the case of static networks, Gillespie algorithms allow massive speedup relative to the random selection approach. This is not the case for the node dynamics in our temporal network model, where a Gillespie event sequence affords very little speedup. Here, the edge activity sequence is a bottleneck, since it still requires $|\mathcal{E}|dt/\langle\tau\rangle$ edge updates per dt on average. As such, it is not for the benefits to speed that we use a Gillespie type event sequence for node updates, it is to account for arbitrarily large spikes in node transmission rates, particularly under the SI rule for temporal networks.

Inverse transform sampling of ψ and Ψ

In this section we discuss the numerical pipeline used to simulate renewal processes, a procedure that amounts to accurately sampling from the interevent time distributions $\psi(\tau)$ and $\Psi(\tau)$. Due to the large values of the standard deviation σ_τ to be examined in this work, currently available software could not be used to sample values of τ. While the excellent `<random>` library for C++ allows rapid sampling from the lognormal, Weibull and gamma distributions, it appears to become inefficient for large σ_τ, especially for the gamma distribution. In any case, directly sampling from Ψ, which often involves special functions, is beyond the scope of this library. Because of this, we build our own sampling routine.

We favour an inverse transform sampling technique, where random values are sampled on an interval $(0, 1)$, and evaluating the inverse of a cdf at this point provides a sample of the underlying pdf. That is, for a random variable $x \in (0, 1)$, evaluating $\Psi^{-1}(x)$ provides a sample τ value from ψ. Further, random sampling of the residual

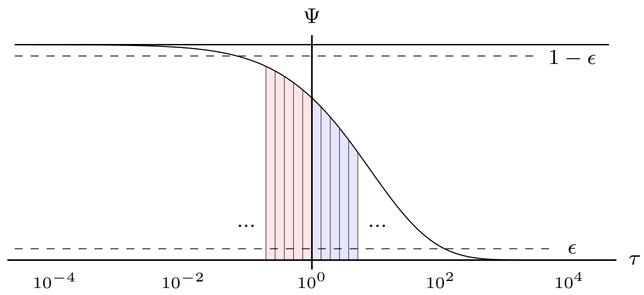

Supplementary Figure 8. **Sampling probability distributions.** Numerical construction of Ψ, a probability density function that can be accurately evaluated at any point τ, at some cost, for all distributions used in this work. This grid results from iterating outwards from $\tau = \langle\tau\rangle = 1$, for increasing τ until $\Psi < \epsilon$, blue grid, and for decreasing τ until $\Psi > 1-\epsilon$, red grid. Inverse transform sampling is then performed on the interval $(\epsilon, 1-\epsilon)$ to provide samples of ψ, using a bisection method on the grid, to find the corresponding τ. A third order spline interpolation on a logarithmic scale provides intermediate values of τ. Additionally, the grid can be cumulatively summed, and inverse transform sampling carried out on the spline of the resulting grid, to provide samples of Ψ.

distribution requires finding the cdf of Ψ, and being able to approximate its inverse.

First, the problem of sampling from ψ is that while Ψ is known, its inverse generally is not. Second, we can't easily sample from Ψ since its cdf is generally unknown to begin with. In fact, we often have enough difficulty simply evaluating Ψ, as is the case when ψ is the gamma distribution, where Ψ has no closed form. As a result, we can only determine Ψ^{-1} approximately. We do this by first generating a grid of Ψ values as shown in Supplementary Fig. 8. Due to the large σ_τ values of interest to us, it is difficult to estimate *a priori* the desired upper and lower limits τ of the grid, which vary significantly depending on the choice of ψ. Said differently, we want ϵ to be as small as possible, in order to allow for the sampling of extreme values of τ, which are critical to the dynamics of our system. Further, it goes without saying that taking the small ϵ limit ensures that $\langle\tau\rangle$ and σ_τ are respected, which is crucial since the latter is a control parameter in our experiment. As shown in Supplementary Fig. 8, we iterate outward from $\tau = \langle\tau\rangle = 1$ uniformly on a logarithmic scale until a desired interval $(\epsilon, 1-\epsilon)$ is obtained. An arbitrary precision library is required to accurately determine Ψ, and we prefer GNU MPFR, a multiple-precision binary floating-point library with correct rounding. See Supplementary Note 8 for details regarding the distributions themselves.

With such a grid accurately computed, we carry out third-order spline interpolation on a logarithmic scale to allow rapid evaluation at arbitrary points τ within the grid. As a first application, the spline can be used to rapidly solve $\Psi(\tau) = x$ using a bisection technique. This provides samples τ of ψ. The grid can then be cumulatively summed to provide the cdf of Ψ over the same domain. Performing approximate inverse transform sampling on the resulting grid returns samples τ of Ψ.

It is worthwhile mentioning that rejection sampling techniques appear to be out of the question here. This is due to the extreme bounding values of τ necessary for the standard deviations σ_τ studied in our choice of heavy-tailed distribution. Even for clever choices of enveloping functions for ψ and Ψ, it is likely that the acceptance rates will be prohibitively low. Finding such functions remains an interesting challenge, but since our bisection approach converges exponentially quickly, it is hard to imagine that a choice of envelope exists that makes rejection sampling faster.

Finally, we comment on experiments involving very large standard deviations σ_τ. Here, not even large networks running for a long time provide an unbiased sample of ψ. Following the central limit theorem, the standard deviation of the sampled mean interevent time equals $\sigma_\tau/\sqrt{n_s}$, if n_s is the number of samples in question, which for the sake of argument is on the order of the number of edges in the network. High quality experimental results can be obtained by simulating large networks, or by averaging over realisations. Note further that large σ_τ experiments in our model happen to coincide with very long simulation times. As a result, even for large σ_τ there is little noise in our results due to the substantial runtime. Finally, ξ_E plays a very important role, and amounts to the fraction of samples of Ψ that are larger than η. A consequence of this is that for large σ_τ, most edges don't even participate, having drawn residual times at $t = 0$ that are longer than the duration of the experiment, determined by $\rho = 1 - e^{-pt}$.

Supplementary Note 5. Laplace transform inversion

In the following sections we describe the numerical pipeline for obtaining the rates μ_j and ν_j, as well as the edge-state distribution E_j. Although these values could be calculated by hand for the case of the exponential distribution, and maybe even the gamma distribution as its Laplace transform is known, we would like to be able to do this numerically for arbitrary $\psi(\tau)$, including those for which the Laplace transform of ψ and Ψ are not known.

These rates are expressed in terms of convolutions. Since we are interested in j-th order convolutions, for arbitrary positive integers j, we prefer to perform products in frequency space, as permitted by the Laplace transform. A j-th order convolution for large j increases exponentially in complexity, and can only be performed directly for very small j. Further, E_j can be broad when σ_τ is large. The Laplace transform is defined as

$$\hat{f}(s) = \mathcal{L}\{f\}(s) = \int_0^\infty e^{-st} f(t)dt, \quad (27)$$

where f is a real-valued function of time t, and $\hat{f}$ a com-

plex valued function of the complex variable $s = \sigma + i\omega$. For the Gaver-Stehfest algorithm in the following section, s is always real, so we can set $\omega = 0$ and write

$$\hat{f}(s) = \int_0^\infty e^{-\sigma t} \cos \omega t f(t) dt - i \int_0^\infty e^{-\sigma t} \sin \omega t f(t) dt. \tag{28}$$

This is necessary when the Laplace transform of f is not know, and must be evaluated numerically, as is the case for the lognormal and Weibull distributions, used throughout this work. There exists a useful framework that unify these different algorithms [2], that express each approach in terms of a weighted sum of $\hat{f}$ values. We shall see that the computation of the forward Laplace transform is the bottleneck, as opposed to finding the weights. As such, the preferred method depends on how many numerical inversions of f are required. This is $2M$ in the Gaver-Stehfest algorithm, $2M+1$ in the Euler algorithm and M in the Talbot algorithm, with M controlling the desired accuracy (see [2] for details). However, only the real integral need be found in the Gaver-Stehfest algorithm, which we prefer for this reason.

Gaver-Stehfest algorithm

We assume a Laplace transform $\hat{f}$ is known, and that we wish to recover the origin unknown function f. In our case, f corresponds to convolutions of ψ and Ψ. For any $t > 0$ and positive integer M, so-called Salzer summation yields the Gaver-Stehfest inversion formula,

$$f(t, M) = \ln(2) t^{-1} \sum_{k=1}^{2M} \zeta_k \hat{f}(k \ln(2) t^{-1}) \tag{29}$$

where the weights ζ_k are given by

$$\zeta_k = \frac{(-1)^{M+k}}{M!} \sum_{j=\lfloor (k+1)/2 \rfloor}^{\min(k,M)} j^{M+1} \binom{M}{j} \binom{2j}{j} \binom{j}{k-j}. \tag{30}$$

Conveniently, the weights ζ_k are independent of the transform being inverted. This means that after a precision M is chosen, weights can be stored in a vector ζ of dimension $2M$ to be reused for a number of transforms $\hat{f}$. We discuss the numerical implementation further in following sections, however mention here that we use the GNU Multiple Precision arithmetic library GMP for ζ_k, in particular its integer summand, and the related MPFR library for floating point arithmetic for manipulating $\hat{f}$. Regarding the weights ζ_k, a useful property for benchmarking is the fact that for all $M \geq 1$,

$$\sum_{k=0}^{2M} \zeta_k = 0, \tag{31}$$

due to the $(-1)^{M+k}$ factor in the definition of ζ_k, these weights oscillate around zero. The Gaver-Stehfest algorithm requires a system precision of approximately $2.2M$ tracked bits, which we input to the constructors of variables in MPFR.

Numerical pipeline

We now describe specifically how the inversion procedure in the previous section can be used to determine the values E_j, μ_j and ν_j described in preceding sections. For simplicity, we refer to these quantities in this section using vectors E, μ and ν, whose dimension is determined by the size of the edge state space n in our master equation, which need not be determined *a priori*. Since E_0 and μ_0 are special cases of E_j and μ_j, we write $E = (E_1, E_2, \ldots, E_n)^T$ for the edge state distribution, and $\mu = (\mu_1, \mu_2, \ldots, \mu_n)^T$ and $\nu = (\nu_1, \nu_2, \ldots, \nu_n)^T$ for positive and negative edge transitions. The function $f(t, M)$ in the Gaver-Stehfest algorithm corresponds to $E_j(\eta, M)$, $\mu_j(\eta, M)$ and $\nu_j(\eta, M)$, where M as before is a parameter of the Gaver-Stehfest algorithm that tunes the accuracy of the approximation.

Calculating the distribution E, and the rate vectors μ and ν amounts to three separate matrix vector products. We require the vector $\boldsymbol{\zeta}$, of dimension $2M$, as per the Gaver-Stehfest algorithm whose k-th element is given by Supplementary Eq. (30), and the Laplace transforms of ψ and Ψ evaluated at $s = k \ln(2) t^{-1}$, denoted ψ_k and Ψ_k, as per Supplementary Eq. (12). We define n as the maximum edge state, which can be as large as one likes here, it is limited only by M, which must be increased for increasing n. These quantities are then used to populate the $n \times 2M$ dimensional matrices $\hat{E}$, $\hat{\mu}$ and $\hat{\nu}$, whose jk-th elements are given by

$$[\hat{E}]_{jk} = \hat{\Psi}_k \cdot \hat{\psi}_k^{j-1} \cdot \hat{\Psi}_k \tag{32a}$$

$$[\hat{\mu}]_{jk} = \hat{\Psi}_k \cdot \hat{\psi}_k^{j} \tag{32b}$$

$$[\hat{\nu}]_{jk} = \hat{\Psi}_k \cdot \hat{\psi}_k^{j-1}, \tag{32c}$$

with $1 \leq j \leq n$ and $1 \leq k \leq 2M$. Determining the distribution of edge states E, and the edge transition rates μ and ν, then amounts to the matrix-vector product of $\hat{E}$, $\hat{\mu}$ and $\hat{\nu}$, respectively, with $\boldsymbol{\zeta}$, such that $E = \hat{E}\boldsymbol{\zeta}$, $\mu = \hat{\mu}\boldsymbol{\zeta}$, and $\nu = \hat{\nu}\boldsymbol{\zeta}$. These quantities are calculated once at the start of the Runge-Kutta solution of the corresponding system, and don't change otherwise. Note that results have to be scaled by $\ln(2)t^{-1}$ as per the definition of $f(t, M)$ in the Gaver-Stehfest algorithm, and μ_j and ν_j normalised by E_j, to adhere to their definitions. Examples of μ and ν values are provided in Supplementary Fig. 6 for the case of a gamma distribution ψ.

Implementation of the Gaver-Stehfest algorithm in C++ created confusion for some time, not because of the calculation of the weights ζ_k, but in its product with $\hat{f}$. The authors of [2] state that while it is clearly required for

the weights ζ_k, arbitrary precision is not required for handling $\hat{f}$. However, we find that double precision is not sufficient in general for $\hat{f}$, which can be confirmed by using double values in C++, or equivalently, setting a precision of 53 bits in a arbitrary precision library. We use a combination of the GMP library for the binomial coefficients, factorial, and exponential in Supplementary Eq. (30). Division is performed after conversion to MPFR. Similarly, $\hat{f}$ in Supplementary Eq. (29) is determined using MPFR, whether the Laplace transform is known in closed form, or if it is calculated from its integral definition.

Supplementary Note 6. Static network topology

In this supplementary note we further consider the effect of network structure on the temporal dynamics outlined in our framework. Our master equation assumes a configuration-model network, whereby network structure is maximally random apart from the degree distribution, which is fixed. We have shown using the relative threshold model that degree distribution, beyond its mean, has little impact on diffusion dynamics in the presence of uncorrelated temporal fluctuations. This is evidenced by the degeneracy of ρ_f values obtained when varying the degree standard deviation in a lognormal degree distribution, while keeping the mean degree fixed. In the following we identify features of network topology that are likely to produce such degeneracy, and those under which the degeneracy may be broken.

We examine the effect of clustering on the dynamics of information cascades [Supplementary Fig. 9(a)]. We do this using an extension to the configuration model due to Newman, where in addition to specifying the number of edges, we specify the number of triangles to which a node is adjacent. In this figure, we interpolate between an 8-regular random network where no triangles are present, and one where all edges form part of a triangle. To do this, we assign a total of 8 stubs to a node, before arbitrarily sorting them into pairs. We assign with probability α the triangle type to a given pair of stubs, constraining them to be wired to another two pairs of triangular stubs, belonging to two other randomly selected nodes. With probability $1-\alpha$, each stub in the pair is assigned the single type. In this way, when $\alpha = 1$, clustering is maximal, and when $\alpha = 0$, clustering is vanishing in the large network limit. For the following, and all experiments in Supplementary Fig. 9, we vary interevent time standard deviation σ_τ of a Weibull interevent time, while fixing its mean to $\langle\tau\rangle = 1$. Memory is set to $\eta = 1$, and node dynamics follow the relative threshold rule with $\phi = 0.15$, in the presence of background noise at rate $p = 2 \times 10^{-4}$.

Like degree distribution, clustering appears to have no impact on information diffusion beyond the Poisson regime, $\sigma_\tau = 1$, where interaction times are random. While burstiness has a decelerative effect beyond this point, it is indifferent to the extent of clustering. This is

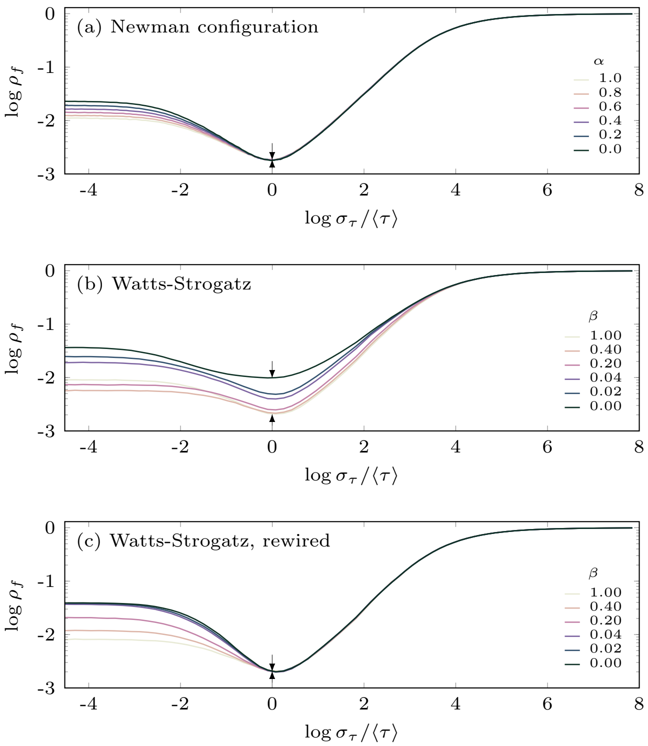

Supplementary Figure 9. **Temporal fluctuations erase local network structure.** (a) Effect of varying the fraction of triangles in a configuration model network, Newman. Average degree is $z = 8$, degree distribution is random regular, α equals 1, no single edges, 0, no triangles in network. (b) The Watts-Strogatz model with $z = 8$. When $\beta = 1$ the network is a ring lattice, 0 approaches a Poisson random graph. (c) Same degree distribution as (b), but maximally random otherwise. Varying degree distribution has no effect on spreading process, only z matters. Local structure is washed away by temporal fluctuations, but global structure, like average path length, remains critical as evidenced by varying β in the Watts-Strogatz model. Nodes follow the relative threshold model with $\phi = 0.15$. Interaction memory is $\eta = 1$. Data is from numerical experiment only, on networks of size 10^5, with 10^2 averages in each case.

in contrast to the when the network is effectively static, such as when $\sigma_\tau = 10^{-4}$, and clustering slows diffusion by a factor of more than 2. The degeneracy in the bursty regime can be understood by accounting for the sparsifying effects of burstiness. That is, at any given time, a triangle is expected to be broken due to one or more edges not recalling an interaction event. As such, active edges produce a subgraph that is locally tree-like with high probability. One way in which this effect can be avoided is if edge activity is correlated, namely, when activity on one edge of a triangle increases the likelihood of activity on the remaining edges. Since burstiness is likely causal in reality, this suggests a promising future research direction.

Since both degree distribution and clustering have proven to be degenerate with respect to uncorrelated in-

teraction events, we depart from the configuration model altogether. By way of contrast, we consider the Watts-Strogatz model, which has the advantage of simultaneously varying local structure, namely clustering and node degree, as well as global structure, in the average path length. By global structure, we mean quantities that only meaningful when taking all nodes in the network collectively. Following typical implementations, we vary the probability β of randomly rewiring edges in an 8-regular ring lattice, thus producing shortcuts to distant nodes in the network. In this way, we interpolate between an entirely regular structure when $\beta = 0$, and a structure close, but not identical to, a maximally random graph when $\beta = 1$. Experimental conditions are identical to those described in Supplementary Fig. 9(a).

We observe a substantial dependence on the rewiring probability β in the Watts-Strogatz model, as evidenced by the non-degeneracy of the curves in Supplementary Fig. 9(b). Small values of β, such as 0.02 and 0.04, are sufficient to substantially reduce the spreading time, at all but the very largest values of $\sigma_\mathcal{T}$. Since small β leaves clustering and degree distribution relatively unchanged, we attribute this accelerative effect to the average path length, which is significantly reduced at small β. In any case, we already expect changes in clustering and degree distribution to have negligible effect on casacade dynamics in the presence of temporal fluctuations, as evidenced by previous experiments. To appreciate the magnitude of the small-world effect, consider that when $\sigma_\mathcal{T} = 1$, for example, burstiness must be increased $\sigma_\mathcal{T} = 30$ to achieve the same effect as reducing β from 1 to 0. In other words, the decelerative effect of the small world topology is as large as that achieved by increasing burstiness by an order of magnitude. We can understand the non-degeneracy here be noting that unlike clustering, average path length is a feature that cannot be undone by temporal fluctuations, except at the largest values when ρ_f approaches 1.

We repeat the experiment performed in Supplementary Fig. 9(b), only now on the corresponding configuration model network, plotting the results in Supplementary Fig. 9(c). The network is initially constructed as before, for identical values of β. Then, we sever all edges and rewire them at random, thus preserving the degree distribution while removing collective structure such as average path length. In this way, degree distribution is shown once again to be degenerate, confirming that the collective structure of the Watts-Strogatz model was responsible for breaking the degeneracy in Supplementary Fig. 9(b).

The above experimental results lead us to conjecture that network features that are essentially collective are most likely to continue to play a role in the presence of temporal fluctuations. This is in contrast to features determined on the level of nodes and their immediate neighbours, such as node degree and clustering. Properties such as average path length, community structure and eigenvector centrality are all examples of manifestly collective structures. In the absence of collective structure, our results in this section highlight the need to explicitly introduced temporal correlations, if properties such as node degree and clustering are to play a role in information diffusion.

Supplementary Note 7. Mean field approximation

In this Supplementary Note we examine the effectiveness of the mean field assumption of edge state transitions. The edge transition rates calculated in this work assume an infinitely large ensemble of stationary, uncorrelated renewal processes. We expect a carefully implemented stochastic temporal network to agree precisely with theory, in the limit of large networks, which is what we confirm. In particular, by viewing the network as a single ensemble of edges, flux measurements indeed show theoretical results for E_j, μ_j and ν_j to be exact on a network wide scale. However, our analysis involves partitioning our network into $2 \times |C|$ classes, namely, an infected and uninfected variant for each of the $|C|$ classes $(\boldsymbol{k}, \boldsymbol{m})$ allowed by the system. Clearly, the densities of nodes over these classes, and the transitions between them are highly dynamic, emptying and filling over the course of a spreading process. Further, transition rates are heterogeneous, particularly with respect to the transmission rate $F_{\boldsymbol{k},\boldsymbol{m}}$ which varies from class to class. The mean field approximation is to assume that edge transition rates for individual classes are the same as for a completely uncorrelated ensemble.

The reason that we expect differences in edge transition rates over these scales is as follows. The emergent rates μ_j and ν_j result from certain assumptions regarding edge statistics at a microscopic level. This is formulated in terms of the history distribution, or distribution of tuples $(\tau_1, \tau_2, \ldots, \tau_j)$ within each η window, on each edge across a given set. This is illustrated in Supplementary Fig. 4. Expressed in these terms, the mean field approach is to assume that the history distribution within a particular class $(\boldsymbol{k}, \boldsymbol{m})$ is completely uncorrelated, and is equivalent to any randomly sampled subset of edges, or indeed the network edge set as a whole. To see how this assumption may break down, consider the flow of uninfected nodes through C as a survival process, whereby a node enters an uninfected class $(\boldsymbol{k}, \boldsymbol{m})$, only exiting and reentering an adjacent uninfected class if it does not become infected in the meantime. To further simplify this picture, consider $(\boldsymbol{k}, \boldsymbol{m})$ to have $F_{\boldsymbol{k},\boldsymbol{m}} = 1$, with neighbouring uninfected classes having $F_{\boldsymbol{k},\boldsymbol{m}} = 0$, as may occur with a threshold model of infection. In such a survival process, it is nodes with a history distribution $(\tau_1, \ldots, \tau_j)$ with short intervals in $(\boldsymbol{k}, \boldsymbol{m})$, that are favourable to survival, exiting to adjacent uninfected classes. History distributions leading to long waiting times in $(\boldsymbol{k}, \boldsymbol{m})$ are more likely to become infected, with survival times following an exponential distribution with mean $F_{\boldsymbol{k},\boldsymbol{m}}$. As such, the history distribution of nodes exiting the class

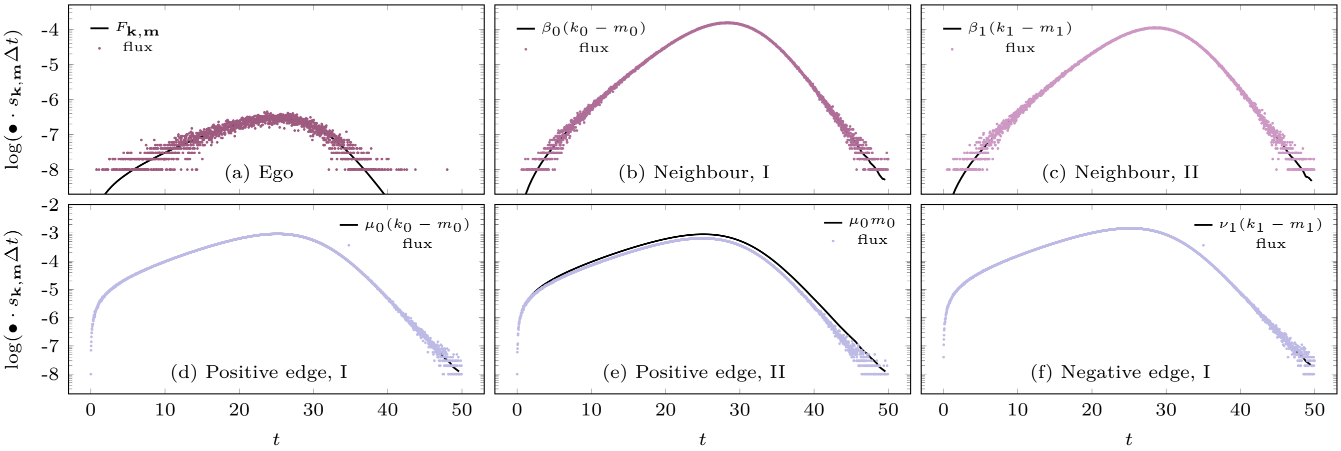

Supplementary Figure 10. **Examination of mean field assumption for class transition rates.** Flux measurement for the set $S_{\boldsymbol{k},\boldsymbol{m}}$ with $\boldsymbol{k} = (2,1)^T$ and $\boldsymbol{m} = (1,0)^T$ during Monte Carlo simulation. Network size is $N = 10^8$, with a 3-regular random degree distribution. Background noise is $p = 2 \times 10^{-4}$, with a relative threshold of $\phi = 0.4$. Interevent time distribution ψ is power law, with $\alpha = 2.5$ and cutoffs $\tau = 1.01$ and 50. Memory duration is $\eta = 1$. Flux is measured over intervals of $\Delta t = 0.02$, shown as points, and compared to the density $s_{\boldsymbol{k},\boldsymbol{m}}$ scaled by the indicated theoretical coefficients, shown as solid lines.

via infections are different to those that survive, and exit via edge or neighbour transitions. Mechanisms like this, and the related effect due to neighbour transitions, gradually transform the history distribution of individual classes, resulting in deviations in the emergent edge transition rates μ and ν. Although the assumption is broken on the scale of individual classes, it is of course preserved when taking all classes together.

We confirm this effect in an experiment whose results are shown in Supplementary Fig. 10. Here, we simulate a spreading process, and carefully record the densities of nodes in each class $(\boldsymbol{k},\boldsymbol{m})$ at all times t, as well the flows between classes over measurement windows of length $\Delta t = 0.02$. To allow Δt to be as small as possible, and approximate the dt in our analytics, we constrain the system to the smallest non-trivial configuration space C. To this end, the degree distribution is 3-regular random, and ψ given by a power law with $\alpha = 2.5$, with lower and upper cutoffs of $\tau = 1.01$ and 50, respectively. By choosing $\eta = 1 < 1.01$, we ensure a two-level edge state space, where edges are in state $j = 0$ or 1. The resulting configuration space has size $|C_3| = 20$, is connected, and resembles a smaller version of Supplementary Fig. 1. By setting network size as large as possible, here $N = 10^8$, the node set is diluted as little as possible over C. We plot the flux measurements of the uninfected class with degree vectors $\boldsymbol{k} = (2,1)^T$ and $\boldsymbol{m} = (1,0)^T$. Node dynamics follow a relative threshold rule with $\phi = 0.4$, and background noise causing infection at a rate $p = 2\times10^{-4}$. As such, the transmission rate for the class in question is $F_{\boldsymbol{k},\boldsymbol{m}} = p$.

Ego transition measurements are shown in Supplementary Fig. 10(a). Since the class initially has density $s_{\boldsymbol{k},\boldsymbol{m}} = 0$, with the network initialised to $\rho = 0$ at $t = 0$, initial measurements show only a handful of ego transitions per Δt up to around $t = 10$, visible here thanks to the logscale. As expected, the rate of ego transition closely agrees with the measured value of $p = 2 \times 10^{-4}$, verified by scaling the total set density by $F_{\boldsymbol{k},\boldsymbol{m}} s_{\boldsymbol{k},\boldsymbol{m}} \Delta t$, given by the solid black curve. This transition is guaranteed to agree with theory if the Gillespie algorithm is correctly implemented, and serves as a useful benchmark in flux measurement experiments. Further, neighbour transition rates are verified in Supplementary Fig. 10(b) and (c), for edges of type $j = 0$ and 1 respectively. Time-dependent rates β_j are calculated using the set of $|C_3| = 20$ empirical densities $s_{\boldsymbol{k},\boldsymbol{m}}$. Scaling $s_{\boldsymbol{k},\boldsymbol{m}}$ for the class in question by $\beta_0(k_0 - m_0)$ and $\beta_1(k_1 - m_1)$ shows remarkable agreement with measured fluctuations.

Flux measurements of positive edge transitions for uninfected and infected neighbours are shown in Supplementary Fig. 10(d) and (e), respectively. While agreement is excellent in (d), a clear deviation emerges in (e). Although the disagreement appears minor on a logarithmic scale, the theoretical μ_0 is off by roughly 20%. The rate of negative edge transition in (f) is in good agreement with theory. A study of the complete state space shows that deviations as in (e) are common, but not systematic, with mean field estimates μ and ν sometimes overestimating, and sometimes underestimating the measured fluxes. The master equation solution of ρ for this experiment, not shown here, miscalculated the overall spreading speed by about 15%. It is likely that the small configuration space here contributed to the error. In much larger systems, this effect is likely diluted, especially since μ and ν do not systematically over or underestimate the class-level edge transition rates. That is, a cancellation effect might emerge.

We confirm that the mean field assumption breaks down due to heterogeneities in transmission rates by studying a purely noise driven variant of the above experiments. That is, $F_{\boldsymbol{k},\boldsymbol{m}} = p$ for all classes $(\boldsymbol{k},\boldsymbol{m})$. The

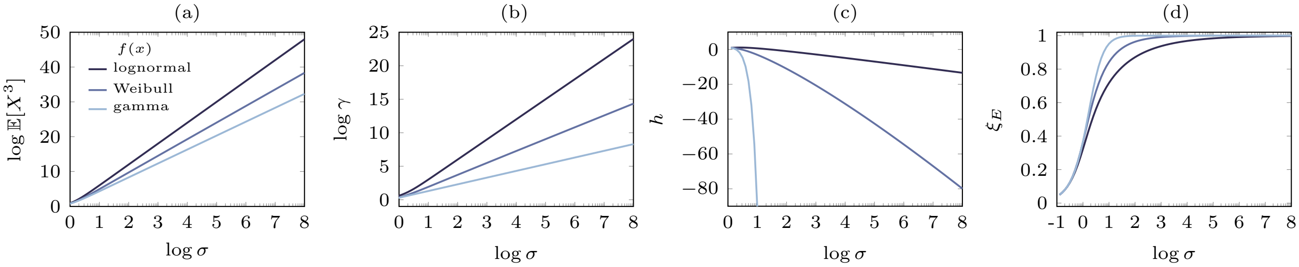

Supplementary Figure 11. **Higher order moments of probability distributions.** Comparing skewness and entropy of probability distributions as a function of standard deviation. Mean is fixed to $\mu = 1$. (a) The third raw moment $\mathbb{E}[X^3]$, and skewness γ in (b), for the lognormal, Weibull and gamma distributions. The third raw moment is the only term that differs in the calculation of skewness. (c) Entropy follows the same trend as (a) and (b). We are interested in the connection between these quantities and ξ_E in (d), the fraction of edges in state $j = 0$, here with $\eta = 1$.

same flux measurements, not shown here, are in perfect agreement with theory in such a setting. Further, the relative set sizes for constant m rows of configuration space are exactly what one would expect given a degree distribution $p(k)$, and a probability distribution E_j that a randomly selected edge is in state j.

Supplementary Note 8. Skewness and entropy

In this Supplementary Note we examine the skewness and differential entropy of the interevent time distributions used in this work, namely the lognormal, Weibull and gamma distributions. Our goal is to understand how these distributions differ after carefully controlling for their mean and standard deviation, μ and σ respectively, and to this end propose skewness and entropy as measures that can be understood as ranking various distributions ψ by the value of their effective sparsity ξ_E.

We control for both the mean and standard deviation when comparing distributions in this work. For a random variable X, this is equivalent to controlling the first and second raw moments, $\mathbb{E}[X] = \mu$ and $\mathbb{E}[X^2] = \mu^2 + \sigma^2$. We wish to examine the differences that remain between our chosen distributions after imposing these constraints. To this end, it is logical to examine the third raw moment $\mathbb{E}[X^3]$. This is usually done by calculating the skewness γ, typically defined as the third standardised moment, or $\mathbb{E}[(X-\mu)^3]$ normalised by σ^3. The normalisation renders the moment scale invariant, meaning the information encoded in the skewness relates only to the "shape" of the distribution in question, and not its variance. Since we're interested in comparing skewness for constant mean and standard deviation, we use the expression

$$\gamma = \frac{\mathbb{E}[(X-\mu)^3]}{\sigma^3} = \frac{\mathbb{E}[X^3] - 3\mu\sigma^2 - \mu^3}{\sigma^3}. \quad (33)$$

Clearly, skewness γ differs from one distribution to another only in the third raw moment $\mathbb{E}[X^3]$, since we keep μ and σ constant. Plotting skewness γ as a function of σ for constant $\mu = 1$ leads to the plot in Supplementary Fig. 11, for the lognormal, Weibull and gamma distributions. The large differences in skewness here correspond to observations regarding effective sparsity ξ_E in earlier parts of this work. As such, the skewness of a distribution provides a good rule-of-thumb for comparing ξ_E for different distributions.

In addition to skewness, we calculate the differential, or information entropy for each distribution used in this work. Differential entropy is defined as

$$h = -\int_0^\infty f(x) \ln f(x) dx, \quad (34)$$

for distributions defined for $x \in (0, \infty)$, as is the case for the lognormal, Weibull and gamma distribution. The motivation for considering the differential entropy is the observation that it was the lognormal distribution, the maximum entropy distribution for which the mean and variance of $\ln(X)$ are specified, that produced the fastest diffusion times in our framework.

As shown in Supplementary Fig. 11, the relative values of skewness and entropy agree qualitatively with expectation. Simply put, the relative values of skewness γ, shown in plot (b), and differential entropy h, shown in plot (c), agree qualitatively with the relative values of effective sparsity ξ_E, shown in plot (d). This supports the rule-of-thumb that the greater the skewness and entropy, the lower the effective sparsity.

Temporal network percolation transition

One of the most striking consequences of interevent time skewness is in the collapse of the giant connected component formed by active edges. In Supplementary Fig. 12 we plot the location of the temporal percolation transition, that is, the point at which active edges can be expected to form giant connected component. There is a sensitive dependence on the choice of interevent time distribution ψ here, as can be seen by comparing the lognormal, Weibull and gamma distributions [Supplemen-

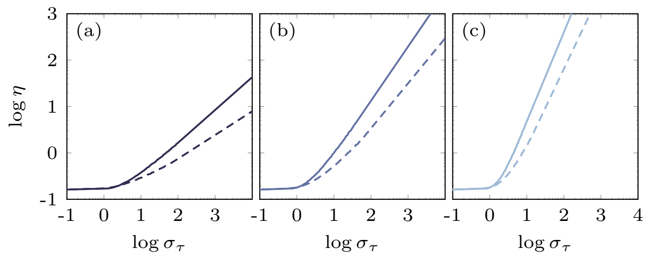

Supplementary Figure 12. **Location of the percolation transition in (σ_τ, η) space.** A giant component formed by the active edges of a temporal network can be expected, at any given time, for the region above the transition. In (a) we illustrate the dependence on memory kernel for a lognormal interevent time distribution. Solid is step-function event memory, dashed is exponential event memory. The corresponding phase transitions for Weibull and gamma interevent time distributions are shown in (b) and (c), respectively. Data was obtained for a network with a lognormal underlying degree distribution, with mean $\langle k \rangle = 7$ and $\sigma_k = 0.5$.

tary Fig. 12(a), (b) and (c), respectively]. Within a single plot, we compare the phase transition under a step-function memory kernel, solid, and exponential memory kernel, dashed.

For all interevent time distributions, an exponential memory kernel delays the collapse of the giant connected component to higher σ_τ values. This delay appears to be more substantial under the lognormal and Weibull distributions, (b) and (c) respectively, than the gamma distribution, (d). Although substantial, the dependence on memory kernel is far exceeded by the dependence on interevent time distribution. We conclude that regardless of the memory kernel, the giant component is fragile under a gamma distribution, robust under a lognormal distribution, and intermediate under a Weibull distribution, relatively speaking.

Supplementary Note 9. Probability distributions

In this Supplementary Note we detail the probability distributions used in this work, in particular those used for the interevent time distribution ψ, and its tail distribution Ψ. For simplicity, the notation used in this Supplementary Note is entirely self contained, and we associate pdfs $f(x)$ with $\psi(\tau)$, and their cdfs $F(x)$ with $1 - \Psi(\tau)$.

Lognormal distribution. The lognormal distribution is defined with respect to an underlying normal distribution with mean $\tilde{\mu}$ and variance $\tilde{\sigma}^2$. These are related to the lognormal mean and variance μ and σ^2 by the relations given in Supplementary Table II, and can be straightforwardly inverted. Provided μ and σ, it has the largest skewness γ of all distributions studied here, as well as the largest differential entropy h. This is not surprising, given that the lognormal can be derived using maximum entropy principles, as discussed in the previous section, and illustrated in Supplementary Fig. 11.

Gamma distribution. The gamma distribution is related to the gamma function, $\Gamma(s)$, described below. The expressions for its mean and variance Supplementary Table II can be easily inverted, resulting in expressions for α and β the provide the desired moments. Note that we set $\mu = 1$ for the temporal network experiments in this work, meaning that when the shape parameter $\alpha = \beta = 1$, coinciding with $\sigma = 1$, we recover the exponential distribution. For values $\alpha < 1$, meaning $\sigma > 1$, the qualitative shape of the exponential is maintained, with an increasingly heavy tail. In contrast, when $\alpha > 1$, meaning $\sigma < 1$, the shape changes, and like the lognormal, f goes to zero in the small x limit. For large α, meaning small σ, the gamma distribution tends to the Dirac delta function. In Supplementary Fig. 11 we observe that the gamma distribution is the least skewed of the distributions considered here, for comparable μ and σ. Entropy is given using ψ, the digamma function, defined in the following section under the Weibull distribution.

Variants of the gamma function. We describe here the necessary approximations in order to numerically construct the cdf of the gamma distribution. Unfortunately, the cdf as stated here is circular, with no closed form expression available. In this section, we discuss a number of series expansions that are necessary to numerically evaluate $\gamma(\alpha, \frac{x}{\beta})$, the lower incomplete gamma function. The gamma function $\Gamma(s)$ is defined, and related to its incomplete variants, as

$$\Gamma(s) = \int_0^\infty t^{s-1} e^{-t} dt \tag{35a}$$

$$= \int_0^x t^{s-1} e^{-t} dt + \int_x^\infty t^{s-1} e^{-t} dt \tag{35b}$$

$$= \gamma(s, x) + \Gamma(s, x). \tag{35c}$$

In other words, the upper and lower incomplete gamma functions are defined by partitioning the integral according to $(0, \infty) = (0, x] \cup [x, \infty)$, given by $\gamma(s, x)$ and $\Gamma(s, x)$, respectively. Sampling from the lower incomplete gamma function will be crucial when initialising our temporal network system at steady state. The choice of series approximation for the lower-incomplete gamma function depends on the shape parameter α, and in turn the standard deviation σ. For small α, meaning large σ, we use

$$\gamma(s, x) = e^{-x} x^s \Gamma(s) \sum_{n=0}^{\infty} \frac{x^n}{\Gamma(s + n + 1)}. \tag{36}$$

This well known approximation, although efficient for large values of standard deviation, becomes prohibitively slow for very large values of α, meaning very small values of σ. At this scale, specifically when $\alpha > 1$ and $\sigma < 1$, we

Supplementary Table II. **Properties of probability distributions.** Properties of the two-parameter probability distributions used to model interevent time in this work. For each we note the probability density function $f(x)$, the cumulative density function $F(x)$, the mean μ and variance σ^2, as well as the third moment $\langle x^3\rangle$, skewness γ, and differential entropy h.

	lognormal*	Weibull†	gamma‡
$f(x)$	$\frac{1}{x\sqrt{2\pi\tilde{\sigma}^2}}\exp\left[-\frac{(\ln x-\tilde{\mu})^2}{\sqrt{2\pi\tilde{\sigma}^2}}\right]$	$\frac{\alpha}{\beta^\alpha}x^{\alpha-1}e^{-(x/\beta)^\alpha}$	$\frac{1}{\Gamma(\alpha)\beta^\alpha}x^{\alpha-1}e^{-x/\beta}$
$F(x)$	$\frac{1}{2}+\frac{1}{2}\mathrm{erf}\left(\frac{\ln x-\tilde{\mu}}{\sqrt{2\tilde{\sigma}^2}}\right)$	$1-e^{-(x/\beta)^\alpha}$	$\frac{1}{\Gamma(\alpha)}\gamma(\alpha,x/\beta)$
μ	$\ln\left(\frac{\mu^2}{\sqrt{\mu^2+\sigma^2}}\right)$	$\beta\Gamma(1+1/\alpha)$	$\alpha\beta$
σ^2	$\ln\left(\frac{\mu^2+\sigma^2}{\mu^2}\right)$	$\beta^2\left[\Gamma(1+2/\alpha)-\Gamma^2(1+1/\alpha)\right]$	$\alpha\beta^2$
$\langle x^3\rangle$	$\frac{(\mu^2+\sigma^2)^3}{\mu^3}$	$\beta^3\Gamma(1+3/\alpha)$	$\mu^3+3\mu\sigma^2+\frac{2\sigma^4}{\mu}$
γ	$\frac{3\mu^2\sigma+\sigma^3}{\mu^3}$	-	$\frac{2\sigma}{\mu}$
h	$\tilde{\mu}+\ln(2\pi e\tilde{\sigma}^2)$	$(1-1/\alpha)\,\gamma_e+\ln(\beta/\alpha)+1$	$\alpha+\ln(\alpha\Gamma(\alpha))+(1-\alpha)\psi(\alpha)$

$*$ Note that the mean and variance correspond to that of the underlying normal distribution, which we denote $\tilde{\mu}$ and $\tilde{\sigma}^2$, respectively.
† We omit skewness since it doesn't simplify like the other distributions. Further, γ_e is the Euler-Mascheroni constant.
‡ Here ψ is the so-called digamma function, defined in the text.

approximate the lower-incomplete gamma function as

$$\gamma(s,x)=e^{-x}x^s\sum_{n=0}^{\infty}\frac{x^n}{s^{\overline{n+1}}} \tag{37}$$

which can be computed recursively using a small number of multiplication and division operations at each step. Unfortunately this approximation fails for large σ, and must be only be used in the small σ, which we do purely for efficiency, since the preceding approximation converges everywhere. Here, $s^{\overline{n+1}}$ is the Pochhammer symbol.

Weibull distribution. Closely related to the gamma distribution is the Weibull distribution. As can be seen in Supplementary Table II, the Weibull distribution cannot be easily parameterised by its mean and standard deviation, as was the case for the lognormal and gamma distribution. The tuple (α,β) providing desired mean and standard deviation can be found using gradient descent. Note that like the gamma distribution, when $\alpha=\beta=1$, we recover the exponential distribution. In the same way as the gamma distribution, the α parameter controls the shape, with large α tending towards the Dirac delta function, and small α an increasingly right skewed, heavy tailed form.

Tuning parameters using gradient descent. We could not find a reference to address the problem of parameterising the Weibull distribution by its mean μ and standard deviation σ. That being the case, we find the values of α and β giving the desired μ and σ using gradient descent. In the following calculations, we actually use the variance denoted by $\nu=\sigma^2$ for simplicity, given its form as expressed in Supplementary Table II. We find α and β by locating the minimum of the loss surface defined by the function

$$l(\mu_i,\nu_i)=\left(\frac{\mu_i-\mu}{\mu}\right)^2+\left(\frac{\nu_i-\nu}{\nu}\right)^2, \tag{38}$$

where $\mu_i=\mu_i(\alpha_i,\beta_i)$ and $\nu_i=\nu_i(\alpha_i,\beta_i)$ are the values of the mean and variance at the i-th step of the procedure, and are functions of α_i and β_i as per Supplementary Table II. Consequently, μ_i and ν_i, as well as α_i and β_i, are treated as continuous variables here. The values μ and ν are the target, and are considered constant in the following. Due to the wide range of σ values sampled, it is crucial to normalise the relative error in each term, by μ and ν respectively. This is because ν varies over orders of magnitude, while μ remains fixed. For the same reason, the displacement at each step of the algorithm is determined on logarithmic scales. We perform this optimisation once, and store the resulting (μ,ν,α,β) tuple in a table for reuse.

The gradient of $l(\mu_i,\nu_i)$ is

$$\nabla l(\mu_i,\nu_i)=\partial_\alpha l(\mu_i,\nu_i)\hat{\boldsymbol{\alpha}}+\partial_\beta l(\mu_i,\nu_i)\hat{\boldsymbol{\beta}}, \tag{39}$$

with α and β components given by

$$\partial_\alpha l(\mu_i, \nu_i) = 2\left(\frac{\mu_i - \mu}{\mu}\right)\frac{1}{\mu}\partial_\alpha \mu_i + 2\left(\frac{\nu_i - \nu}{\nu}\right)\frac{1}{\nu}\partial_\alpha \nu_i \tag{40}$$

and

$$\partial_\beta l(\mu_i, \nu_i) = 2\left(\frac{\mu_i - \mu}{\mu}\right)\frac{1}{\mu}\partial_\beta \mu_i + 2\left(\frac{\nu_i - \nu}{\nu}\right)\frac{1}{\nu}\partial_\beta \nu_i, \tag{41}$$

with partial derivatives

$$\partial_\alpha \mu_i = -\frac{\beta}{\alpha^2}\Gamma(1+1/\alpha)\psi(1+1/\alpha), \tag{42}$$

$$\partial_\alpha \nu_i = -\frac{2\beta^2}{\alpha^2}\left[\Gamma(1+2/\alpha)\psi(1+2/\alpha) - \Gamma^2(1+1/\alpha)\psi(1+1/\alpha)\right], \tag{43}$$

and

$$\partial_\beta \mu_i = \Gamma(1+1/\alpha), \tag{44}$$

$$\partial_\beta \nu_i = 2\beta\left[\Gamma(1+2/\alpha) - \Gamma^2(1+1/\alpha)\right], \tag{45}$$

respectively. Here ψ is the so-called digamma function, which is also required for the calculation of the entropy of the gamma distribution, defined in Supplementary Table II. It is defined as the logarithmic derivative of the gamma function, or

$$\psi(z) = \frac{d}{dz}\ln(\Gamma(z)) = \frac{\Gamma'(z)}{\Gamma(z)}. \tag{46}$$

It can be found using a simple series approximation,

$$\psi(z) = -\gamma_e + \sum_{n=0}^{\infty}\left(\frac{1}{n+1} - \frac{1}{n+z}\right), \tag{47}$$

for $z \neq -1, -2, -3, \ldots$, where γ_e here denotes the Euler-Mascheroni constant. Although the series is infinite, in practice it converges quite rapidly, even when applying strict thresholds. Along with an inputted step size, this provides the next point $(\mu_i, \nu_i, \alpha_i, \beta_i)$ in the procedure. Adaptive step size is incorporated in gradient descent, where step size $h_i = l_i$ if $l_i < 0.1$, and $h_i = 0.1$ otherwise. The gradient is estimated manually by calculating the local neighbourhood of the current point α_i, β_i. Stochastic gradient descent techniques may be incorporated to improve the convergence rate.

Note that a multiple precision library appears to be necessary even when the desired precision in the loss function l not beyond the bounds of default machine precision, such as `double` and `long double`. As always, this induces a cost in terms of computation time, so we calculate all the tuples $(\mu, \sigma, \alpha, \beta)$ that are needed for the entire calculation just once, and then store these in an external lookup table.

Supplementary References

[1] Jo. H.-H., Perotti, J. I., Kaski, K., & Kértesz, J. Analytically solvable model of spreading dynamics with non-Poissonian processes. *Phys. Rev. X* **4**, 011041 (2014).

[2] Abate, J. & Whitt, W. A unified framework for numerically inverting Laplace transforms. *INFORMS Journal on Computing* **18**, 408-421 (2006)
