# Supplementary material for: Dynamics of cascades on burstiness-controlled temporal networks
Source: Nat Commun. 2021 Jan 8;12:133. doi: 10.1038/s41467-020-20398-4 (PMC7794342; doi:10.1038/s41467-020-20398-4)
Supplement: Supplementary file 2 — Peer Review File [file 41467_2020_20398_MOESM2_ESM.pdf]

Reviewer #1 (Remarks to the Author):

The authors develop an analytical framework to analyze the effect of network fluctuations on different variants of spreading on temporal networks. Network fluctuations are modeled as a renewal process and thus characterized by the inter-event time distribution governing the activity of links in the network. They use this to estimate the noise-normalized infection density and its dependence on the time window and the inter-event time distribution. This helps explain what previously appeared to be an inconsistency, namely the acceleration with increased variance, in threshold models. They then show that the differences in the noise-normalized infection density curves across three contagion models, three inter-event time distribution and some different parameter values therein, can largely be attributed to changes in effective sparsity.

Achieving analytic approximations for dynamic network models is difficult and rare, making this contribution inherently interesting. Furthermore their analysis is mathematically careful and thorough and achieves a very good agreement with simulation results. My main concern is that it may be accessible only to a limited number of people in its current form as it draws on deep knowledge from various fields as well as introducing several non-obvious concepts itself in order to achieve its analysis.

I have a few suggestions/questions/comments:
The authors could consider making a figure to illustrate how the network fluctuations are modeled and how and where the burstiness comes in. In particular it took me a while to figure out if the network fluctuation events and the spreading events were the same thing or entirely separate dynamics. (I have come to the conclusion that they are different but it would be interesting to attempt to apply this analysis also for temporal network models where dynamics of and on the network are coupled, such as the adaptive voter model. However, that would probably be out of scope for the current paper.)

The underlying static networks considered here are only determined by their degree distribution. What impact do other characteristics of networks have? In
Chakrabarti, Deepayan, et al. "Epidemic thresholds in real networks." ACM Transactions on Information and System Security (TISSEC) 10.4 (2008): 1-26.
it is for example shown that the Eigenvalues play a large role determining how epidemics spread in static networks, which I guess is related sometimes
Flaxman, A., Frieze, A., & Fenner, T. (2005). High degree vertices and eigenvalues in the preferential attachment graph. Internet Mathematics, 2(1), 1-19.
Chung, F., Lu, L., & Vu, V. (2003). Eigenvalues of random power law graphs. Annals of Combinatorics, 7(1), 21-33.
But certainly not for networks with a peaked degree distribution and a strong community structure, which is relevant for in-person social networks
Molkenthin, N., Schröder, M., & Timme, M. (2018). Adhesion-induced discontinuous transitions and classifying social networks. Physical review letters, 121(13), 138301.
It would be useful to explicitly discuss the topology of the networks used and its implications for applicability in different scenarios.

On page 1 “Epidemic spreading over temporal networks appears to be slowed due to burstiness isome cases [11, 24–26], while accelerated in others” seems to (linguistically) contradict the statement in the abstract “...but can only decelerate epidemic spreading.”

Reviewer #2 (Remarks to the Author):

The authors consider a class of dynamic processes using event times that arrive according to a renewal process (the waiting time distribution between consecutive events has memory) atop a static connectivity structure. From my perspective, the key contribution is to show that, interestingly,

different dynamical processes behave similarly after "accounting for the skewness of different interevent time distributions".

The authors develop a mean-field approximation by considering the proportion of nodes in a particular state (infected, not infected) conditional on the attributes of the neighbourhood (e.g. number of neighbours with a particular number of interactions in the past timespan) of said nodes. Whilst a neat approach, the manuscript is rather difficult to read as the main text refers to the methods section at the end of the document which in turn refers to the supplementary material which in turn refers to the main text. Restructuring the manuscript to achieve a more linear flow through the argument would greatly help its comprehensibility.

Crucially, the dynamics do not only depend on the interevent time distributions (nicely explored in the document), but also the memory kernel. In this manuscript, the memory kernel is chosen to be a step function because it is "the simplest such coupling". It would be interesting to better understand:
(a) the criteria used to evaluate the simplicity of the coupling, e.g. human intuition or mathematical tractability? Depending on one's approach, one might argue that an exponential memory kernel is "more simple" from a mathematical perspective, but it would give rise to different dynamics.
(b) the motivation for using a step function beyond mathematical tractability in the mean-field approximation. For example, do step-like functions of memory commonly appear in real-world systems?

The authors discuss or hint at a number of interesting scientific questions:
(a) "acceleration due to burstiness appears to be a hallmark of threshold mechanisms"--can this conjecture be tested using the methods developed in the manuscript?
(b) "It remains to determine why the value of ξ_E is so sensitive to the choice of interevent time distribution ψ, and in particular, what the properties are of a given distribution ψ that most contribute to the value of ξ_E"--first, the question of sensitivity to ψ can be seen in equation (2): ξ_E is a functional of ψ. Second, exploring the properties of the interevent time distribution that most affect ξ_E could yield valuable insights and could strengthen the scientific contributions of the manuscript.

I would be keen to read the manuscript a second time after these high-level comments have been addressed. In particular, providing more context on the modelling choices (e.g. in terms of motivating examples) and answering some of the scientific questions raised in the manuscript, could make the manuscript appeal to a broader audience compared with its current leaning towards method development.

Some minor comments:
* Using a wider range of symbols could improve readability (as opposed to using bold/normal font to denote different entities).
* It would be nice to elaborate on other conjectures. For example, the authors write "flux measurements of these transitions [...] are expected to be exact", but do not explain why one should have this expectation. They note that "it is possible to show that [...] the size of C behaves like $O(k^{2n})$". A reference to the supplementary material with more context would be nice.

Reviewer #3 (Remarks to the Author):

This paper investigates the problem of modeling temporal networks with bursty interactions.
The proposed model is used mainly to understand the dynamics of information diffusion under some (well-established) mechanisms (i.e., threshold-based information diffusion, both relative and absolute, as well as Susceptible-Infected epidemic spreading).

The paper deals with and relevant problem and provides strong contributions.

Here are some comments that, if addressed properly, may further strengthen the paper:
- The relationship with related work focusing on burstiness in temporal networks (references listed in the Introduction) should be better stated. This would make the reader better appreciate novelty and contributions of the paper.
- A critical fair evaluation of the proposed model, highlighting both strengths and limitations, is currently missing. It would be nice to have it, so as to better understand how realistic the proposed model is and how the paper can pave the way for further research.
- Another important deficiency is a discussion of the potential applications of the proposed model.
- Finally, it would be nice to understand how the proposed model is tied to the considered mechanisms of information diffusion, or it can be easily extended to other mechanisms.

Reviewer #1 (Remarks to the Author):

We thank the Reviewer for their positive evaluation of our work, in particular recognising that our contribution is “difficult and rare” and “inherently interesting”. Below we address all comments, which we found to be helpful and constructive, and highlight the corresponding changes in the manuscript. We hope that the Reviewer will find our revised manuscript suitable for publication in Nature Communications.

Comment 1. The authors could consider making a figure to illustrate how the network fluctuations are modelled and how and where the burstiness comes in. In particular it took me a while to figure out if the network fluctuation events and the spreading events were the same thing or entirely separate dynamics. (I have come to the conclusion that they are different but it would be interesting to attempt to apply this analysis also for temporal network models where dynamics of and on the network are coupled, such as the adaptive voter model. However, that would probably be out of scope for the current paper.)

The Reviewer’s interpretation of our model is indeed correct, fluctuations in interaction events (edge dynamics) occur independently of the diffusion process (node dynamics). Changes to node state occur passively, as a function of the state of its neighbours, along with the state of adjacent edges. To make this evident for the reader, we have made an illustrative figure (Fig.1 in the revised manuscript) that we hope clarifies this point. Further, the idea of coupled edge and node dynamics is indeed within the scope of our framework, which we now allude to in our discussion of applications and future work.

Regarding the question of accessibility of our work, we have extensively revised the introduction by explaining where our contribution fits in the existing literature. Further, the motivation behind our modelling is now better grounded in real world examples. Finally, we plainly evaluate the strengths and limitations of our work, in addition to its potential applications. We hope that together, these changes increase the accessibility of our work.

Comment 2. The underlying static networks considered here are only determined by their degree distribution. What impact do other characteristics of networks have? In

- Chakrabarti, Deepayan, et al. "Epidemic thresholds in real networks." ACM Transactions on Information and System Security (TISSEC) 10.4 (2008): 1-26.

it is for example shown that the eigenvalues play a large role determining how epidemics spread in static networks, which I guess is related sometimes

- Flaxman, A., Frieze, A., & Fenner, T. (2005). High degree vertices and eigenvalues in the preferential attachment graph. Internet Mathematics, 2(1), 1-19.
- Chung, F., Lu, L., & Vu, V. (2003). Eigenvalues of random power law graphs. Annals of Combinatorics, 7(1), 21-33.

But certainly not for networks with a peaked degree distribution and a strong community structure, which is relevant for in-person social networks

- Molkenthin, N., Schröder, M., & Timme, M. (2018). Adhesion-induced discontinuous transitions and classifying social networks. Physical review letters, 121(13), 138301.

It would be useful to explicitly discuss the topology of the networks used and its implications for applicability in different scenarios.

As the Reviewer remarked, the underlying network topology was indeed the least explored component of our model. To better address the role of network structure, we argue in our updated manuscript that the sparsifying effects of

temporal fluctuations break down local structure, while leaving collective structure intact. By local structure we mean graph properties that are determined entirely on the scale of individual nodes and their immediate neighbours, such as node degree and clustering. Collective structure, in contrast, refers to the family of properties that can only be defined by considering nodes in their ensemble, such as average path length, modularity and centrality, among others. In the presence of temporal interactions, the underlying degree distribution does not determine dynamical outcomes. This can be seen in Fig. 2(a) [previously Fig. 1(a)] of the main text, where, ρ_f does not vary as a function of degree standard deviation (in the bursty regime).

Based on this observation, we examine the effect of varying collective, rather than local, structure. In a new section in the SI (Supplementary Note 6) we perform extensive experiments over the Watts-Strogatz model. In this way we are able to show that collective properties, here average path length, continue to play a substantial role, whereas clustering and degree distribution do not. We benchmark experiments on the Watts-Strogatz model against Newman's configuration model with triangles, and configuration model rewiring of the Watts-Strogatz model. Although we have not necessarily used the language of eigenvalues and eigenvectors, as mentioned by the Reviewer, graph spectra indeed exemplify what we mean by collective structure. The results of this supplementary note are used to strengthen the conclusion drawn in the main text.

One implication of this, as we mention in the revised manuscript, is that correlations between temporal edge activity must be introduced in order for underlying local structure to play a role. Since there is evidence that burstiness is indeed causal, this suggests a promising future direction.

Comment 3. On page 1 "Epidemic spreading over temporal networks appears to be slowed due to burstiness in some cases [11, 24–26], while accelerated in others" seems to (linguistically) contradict the statement in the abstract "...but can only decelerate epidemic spreading."

We have revised this paragraph in the introduction, and better explain the different dynamical scenarios.

Reviewer #2 (Remarks to the Author):

First, we thank the Reviewer for their positive and very constructive comments. Below we address all the comments while we highlighted the corresponding changes in the manuscript. We hope that the Reviewer will find our revised manuscript suitable for publication in Nature Communications.

Comment 1. Whilst a neat approach, the manuscript is rather difficult to read as the main text refers to the methods section at the end of the document which in turn refers to the supplementary material which in turn refers to the main text. Restructuring the manuscript to achieve a more linear flow through the argument would greatly help its comprehensibility.

We understand that the Reviewer has found our manuscript difficult to read, and we have taken this very seriously. Since presentation was also a concern of Reviewer #1, we have paid particular attention to the restructuring of our revised manuscript.

Regarding the flow of the main text, the Methods section, and the supplementary material (SI), we identified the following problems. First, there was substantial overlap in the Methods and the SI, especially regarding the discussion of the configuration space transitions. This is now contained entirely in the Methods, with the SI reserved for details of the master equation solution besides transitions. Second, we believe that the linearity of the argument was reduced by the order and focus of the experiments in the main text. In Fig. 2 of the original manuscript, we compared transmission rules in depth using heat maps, keeping everything else fixed. Then, in Fig. 3 we compared interevent time distributions, keeping everything else fixed. In our revised manuscript, we consolidate our message by focusing on the temporal dimension. To this end, we explore a single heat map through the lens of varying interevent time distributions (Fig. 2 original, Fig. 4 revised). Rather than being a detour, the heat map study now serves to

strengthen the original interevent time comparison (Fig. 3, original and revised), revealing quantitatively that skewness in τ is behind the variation in dynamical outcomes, and building a common theme among these figures. Finally, we have removed confusing back-references from the SI to the Methods and main text.

It is worth mentioning, too, that the first figure in the revised manuscript is now a schematic diagram of our model, so there is no need to search through the Methods and SI for clarity regarding the model. As discussed in the following comment, we have paid special attention to improving the model motivation, which we hope improves the flow of the argument.

Comment 2. Crucially, the dynamics do not only depend on the interevent time distributions (nicely explored in the document), but also the memory kernel. In this manuscript, the memory kernel is chosen to be a step function because it is "the simplest such coupling". It would be interesting to better understand:
(a) the criteria used to evaluate the simplicity of the coupling, e.g. human intuition or mathematical tractability? Depending on one's approach, one might argue that an exponential memory kernel is "more simple" from a mathematical perspective, but it would give rise to different dynamics.
(b) the motivation for using a step function beyond mathematical tractability in the mean-field approximation. For example, do step-like functions of memory commonly appear in real-world systems?

We thank the Reviewer for these insightful questions. In addressing these concerns, we obtained results that make our results more general, and strengthened our conclusion scientifically.

To address point (a), the mathematical simplicity is due to the interaction window being finite, with width η. To determine key quantities such as the edge state distribution E_j, and edge transition rates μ_j and ν_j, we perform a sum over all possible temporal configurations in the η-window [Supplementary Eqns. (8), (9) and (10)]. Such a window is undefined when the memory kernel is exponential, rather than a step-function. In the exponential case, there is no upper limit to how far back in an edge's history we have to look in order to find active events. The finite window in the step-function case is thus blurred, complicating the sum over configurations.

Although it is not clear how to obtain the analytic properties of an exponential kernel (E_j, μ_j and ν_j), we can perform additional experiments to determine how exponential memory kernels may impact dynamical outcomes. We decided to do this through the lens of the temporal network percolation transition. Interestingly, our conclusions are largely unchanged under an exponential memory kernel. We include a figure in the SI (Supplementary Fig. 12) where we have simulated the stochastic temporal network under an exponential kernel, for lognormal, Weibull and gamma interevent time distributions. In all cases, it is simply the angle made by the phase transition in the (σ_τ, η) plane that changes. The change in angle is such that the giant temporally-connected component is more robust under an exponential kernel. In other words, the degree of burstiness required to collapse the giant component is always larger under an exponential kernel than a step-function kernel.

To address point (b), in our revised manuscript we take special care to outline the motivation for using step-function memory kernels. To do this, we refer to literature on the "attention economy," highlighting that when content is cheap and abundant, cognitive resources such as attention are finite due to physiological limits and are thus precious. One of the ways that digital platforms allow information to compete in the attention economy is by introducing ephemeral content, content that is expressly erased after a specified period of time. Snapchat, Facebook, Instagram, Whatsapp, Twitter and Wechat are all examples of platforms that allow ephemeral content, typically referred to as "stories," that disappear after 24 hours. Information that propagates by means of these interactions are directly applicable to a step-function memory kernel. Even when content is persistent, meaning it isn't erased, it may suffer a sudden drop in visibility due to competing content. The attention economy literature underlines that information platforms display content on a front page that has finite space, with old content eventually making way for the new. In general, platforms prioritise algorithmically recently posted content. Persistent content, therefore, may be better modelled by an exponential memory kernel.

Comment 3. The authors discuss or hint at a number of interesting scientific questions:
(a) "acceleration due to burstiness appears to be a hallmark of threshold mechanisms"--can this conjecture be tested using the methods developed in the manuscript?
(b) "It remains to determine why the value of ξ_E is so sensitive to the choice of interevent time distribution ψ, and in particular, what the properties are of a given distribution ψ that most contribute to the value of ξ_E"--first, the question of sensitivity to ψ can be seen in equation (2): ξ_E is a functional of ψ. Second, exploring the properties of the interevent time distribution that most affect ξ_E could yield valuable insights and could strengthen the scientific contributions of the manuscript.

In response to (a), although empirical experiments allow us to reasonably infer that burstiness may be accelerative for threshold models [as per our data-driven experiments, see Fig. 2(c) and (d), revised manuscript, Fig. 1(c) and (d), original], showing that it is unlikely in epidemic processes is more challenging. That is, to demonstrate the existence of an effect, a single scenario is sufficient. To show that it is unique to threshold-driven information diffusion remains an outstanding challenge.

In the process of responding to the concerns in (b), we discovered that each distribution used to model interevent time in our work is either a special (gamma and Weibull distributions) or limiting (lognormal distribution) case of the so-called generalised gamma distribution. This allows us to drastically simplify the comparison of the interevent time distributions. In our original work we focused on two-parameter distributions parameterised by mean and standard deviation. Since the generalised gamma distribution contains three parameters, two can be parameterised by mean and standard deviation, with the third used to interpolate between the gamma, Weibull and lognormal case. This is enlightening, since it reveals that the results obtained by comparing these distributions [Fig. 4(b) and (e), original, Fig. 3(b) and (e), revised manuscript; along with Fig. 4(a), revised manuscript] are really just three data points in a continuous family of results.

We explore this insight through the lens of the temporal network phase transition [Fig. 4(a), revised manuscript]. Here, alternating between the gamma, Weibull and lognormal distribution serves to change the angle of phase transition in the (σ_τ, η) plane (in much the same way as varying the memory kernel does, as per Supplementary Fig. 12, revised manuscript). Roughly speaking, then, we can smoothly vary the location of the phase transition by tuning the free parameter in the generalised gamma distribution. We do this in Fig. 4(b) of the revised manuscript, parameterising the third degree of freedom by skewness. Although it is difficult numerically to obtain the lognormal from the generalised gamma distribution, it is straightforward to interpolate between the gamma and Weibull case. The extrapolation to the limiting lognormal case is shown as a dashed line in Fig. 4(b), revised manuscript.

One application of this insight is to highlight the usefulness of the generalised gamma distribution when fitting models to empirical data. Indeed, it is often used to determine which of the lognormal, gamma and Weibull distributions are best suited to a given dataset. Our work shows that this choice can be of utmost importance. For a given value of interevent time mean and standard deviation, the resultant temporal network may either be above percolation, below percolation, or at criticality, with no way of determining which. Our revised manuscript explains that one additional measurement, such as interevent time skewness, is sufficient to discriminate between these cases, and thus characterise the temporal connectivity. And of course, temporal connectivity broadly determines the outcome of information diffusion.

Minor comment. Using a wider range of symbols could improve readability (as opposed to using bold/normal font to denote different entities).

We agree with the Reviewer that a wider range of symbols could somewhat improve the presentation of the formal description, however we are bound to adhere to the current notation due to an increasing number of papers using this

notation. Nevertheless, we added a new schematic figure (Fig. 1 of the revised manuscript), to give explicit examples of k, k_j and **k** in order to increase readability.

Minor comment. It would be nice to elaborate on other conjectures. For example, the authors write "flux measurements of these transitions [...] are expected to be exact", but do not explain why one should have this expectation. They note that "it is possible to show that [...] the size of C behaves like $O(k^{2n})$". A reference to the supplementary material with more context would be nice.

We have updated the Methods and SI to better explain these important points.

In summary, flux measurements of ego transitions are guaranteed to agree with their theoretical value of $F_{k,m}$, on the condition that Monte Carlo simulation has been done correctly. Ego transitions drive the dynamical process, and since node dynamics are Markovian, node infection occurs as a Poisson process. That is, a node with configuration (k,m) becomes infected at a constant rate $F_{k,m}$, which we ensure by drawing a waiting time to infection from an exponential distribution with mean $1 / F_{k,m}$. In fact, we verify that node dynamics have been implemented correctly using flux measurements - in the large network limit, ego transitions from class (k,m) *must* agree with $F_{k,m}$, or something has gone wrong in our implementation.

Regarding the asymptotic growth of configuration space C, we have now included in Supplementary Note 1 the analytical estimates used to arrive at the result of $\Theta (k^{2n})$. This result is verified by numerically constructing C over a wide range of values of k and n. This comparison forms Supplementary Fig. 3.

Reviewer #3 (Remarks to the Author):

First, we thank the Reviewer for their positive and very constructive comments, in particular recognising that our “paper deals with a relevant problem and provides strong contributions.” Below we address all the comments while we highlighted the corresponding changes in the manuscript. We hope that the Reviewer will find our revised manuscript suitable for publication in Nature Communications.

Comment 1. The relationship with related work focusing on burstiness in temporal networks (references listed in the Introduction) should be better stated. This would make the reader better appreciate the novelty and contributions of the paper.

We thank the referee for identifying how the presentation might be improved in terms of accessibility. We have carefully rewritten the introduction of the revised manuscript to better contextualise our work in relationship with the broad literature of bursty dynamical systems. We hope that after these changes our contribution will be more easily identified.

Comment 2. A critical fair evaluation of the proposed model, highlighting both strengths and limitations, is currently missing. It would be nice to have it, so as to better understand how realistic the proposed model is and how the paper can pave the way for further research.

We have fairly outlined the strengths and weaknesses of our framework in the second paragraph of the revised discussion. In summary, the biggest drawback is the exponential growth in the size of the master equation system, $\Theta (k^{2n})$. Thus, researchers using this framework will become aware that as their system size increases it is eventually more straightforward to simply perform Monte Carlo simulations rather than solve such a large system of equations. In light of this, we consider the valuable contributions of our study to be the by-products of our analytical contribution. These limitations are now weighed against the numerous positive contributions of our work in the revised discussion.

Comment 3. Another important deficiency is a discussion of the potential applications of the proposed model.

We have revised the discussion to include the potential applications of our work, which we summarise here.

On the theoretical side, we have shown that a master equation framework, Markovian in essence, can be used to well approximate the dynamics of a non-Markovian system. Since our master equation can be viewed as a continuous time Markov chain, it implies the existence of a Markovian stochastic temporal network exhibiting almost identical behaviour to that of the renewal process model. In other words, while edge transition rates μ_j and ν_j are emergent in the case of our renewal process model, they can be viewed as constant-rate transitions in a memoryless system, such as a random walk model of edge state. This provides insight to future modelling of non-Markovian systems, and we now suggest the application of this approach to Hawkes processes.

On the empirical side, our work reveals that dynamical outcomes such as spreading time, and the temporal network percolation transition, exhibit sensitive dependence on the shape of the interevent time distribution. In our revised manuscript, we interpolate between these distributions using the generalised gamma distribution, of which the gamma and Weibull are special cases, and the lognormal is a limiting case. Our work highlights the importance of higher-order properties of interevent time distributions, not just mean and standard deviation. Using our approach, the generalised gamma distribution can account for a third parameter such as skewness, and thus used to estimate dynamic quantities, such as the likelihood of a given empirical interevent time distribution forming a giant connected component.

Comment 4. Finally, it would be nice to understand how the proposed model is tied to the considered mechanisms of information diffusion, or it can be easily extended to other mechanisms.

We have included a new section in Supplementary Note 1, titled "models of binary-state dynamics," where we briefly outline a number of models to which our framework can be straightforwardly applied. These include the SIS model, the language model, and voter models. One of the biggest advantages of a configuration-based analytical solution, is that we can trivially alternate between binary-state models. This is because models of node dynamics are contained entirely in the response function $F_{k,m}$.

Reviewer #1 (Remarks to the Author):

The authors have addressed all my questions to my satisfaction. I find the manuscript more readable with the changes and enjoyed the new results regarding network topology and interevent distributions. I only have a few very minor comments:

On page 2 in the description of the new figure it would be helpful that the degree vector is defined at each node, either in words or as \textbf{k}(u).

On page 3 in the second Paragraph "Generalising this rule to the case or arbitrary interaction strength [33, 47], in the RT model...", i believe or should be of.

Reviewer #3 (Remarks to the Author):

The authors satisfactorily addressed all my comments.

Reviewer #1 (Remarks to the Author):

We thank the Reviewer for their final comments, and are pleased that our changes have been to their satisfaction.

Minor Comment. On page 2 in the description of the new figure it would be helpful that the degree vector is defined at each node, either in words or as \textbf{k}(u).

The degree vectors in this figure are \textbf{k}(u) = (1,0,0,1,0,0,0,0,0,1), \textbf{k}(v_1) = (0,0,0,0,0,0,0,0,0,1), \textbf{k}(v_2) = (1,0,0,0,0,0,0,0,0,0), \textbf{k}(v_3) = (0,0,0,1,0,0,0,0,0,0). Since this is quite cumbersome, we prefer to retain the current sparse approach, where we specify that degree vectors contain zeros except at j = 0, 3 and 9.

Minor Comment. On page 3 in the second Paragraph "Generalising this rule to the case or arbitrary interaction strength [33, 47], in the RT model...", I believe or should be of.

We have now corrected this.
